# Lipid turnover and SQUAMOSA promoter-binding proteins mediate variation in fatty acid desaturation under early nitrogen deprivation revealed by lipidomic and transcriptomic analyses in *Chlorella pyrenoidosa*

**DOI:** 10.3389/fpls.2022.987354

**Published:** 2022-09-29

**Authors:** Rui Wang, Xiaoling Miao

**Affiliations:** ^1^ State Key Laboratory of Microbial Metabolism, School of Life Sciences and Biotechnology, Shanghai Jiao Tong University, Shanghai, China; ^2^ Joint International Research Laboratory of Metabolic & Developmental Sciences, Shanghai Jiao Tong University, Shanghai, China; ^3^ Biomass Energy Research Center, Shanghai Jiao Tong University, Shanghai, China

**Keywords:** microalgae, nitrogen deprivation, fatty acid desaturation, lipid turnover, SQUAMOSA promoter-binding protein

## Abstract

Nitrogen deprivation induces variations in fatty acid desaturation in microalgae, which determines the performance of biodiesel and the nutritional value of bioproducts. However, the detailed scenario and the underlying regulatory mechanism remain unclear. In this study, we attempt to outline these scenario and mechanisms by performing biochemical, lipidomic, and transcriptomic analyses in *Chlorella pyrenoidosa* and functional characterization of transcription factors in *Yarrowia lipolytica*. We found that early nitrogen deprivation dramatically reduced fatty acid desaturation without increasing lipid content. The contents of palmitic acid (16:0) and oleic acid (18:1) dramatically increased to 2.14 and 2.87 times that of nitrogen repletion on the second day, respectively. Lipidomic analysis showed the transfer of polyunsaturated fatty acids from phospholipids and glycolipids to triacylglycerols, and an increase in lipid species with 16:0 or 18:1 under nitrogen deprivation conditions. Upregulated stearoyl-ACP desaturase and oleyl-ACP thioesterase promoted the synthesis of 18:1, but restricted acetyl-CoA supply revealed that it was the intensive lipid turnover instead of an attenuated Kennedy pathway that played an important role in the variation in fatty acid composition under early nitrogen deprivation. Finally, two differentially expressed SQUAMOSA promoter-binding proteins (SBPs) were heterologously expressed in *Y. lipolytica*, demonstrating their role in promoting the accumulation of total fatty acid and the reduction in fatty acid desaturation. These results revealed the crucial role of lipid turnover and SBPs in determining fatty acid desaturation under early nitrogen deprivation, opening new avenues for the metabolic engineering of fatty acid desaturation in microalgae.

## Introduction

As the greenhouse effect intensifies due to greenhouse gas emissions (mainly CO_2_) (Mikhaylov et al., 2020), microalgae have attracted widespread attention for their efficient capacity for CO_2_ biofixation (Daneshvar et al., 2022), along with the production of biomass, biofuel, and bioactive molecules (Siddiki et al., 2022). Microalgae-based biodiesel is a third-generation feedstock; multiple studies have researched its species selection, cultivation, harvesting, and downstream processes over the years to obtain economically feasible biodiesel (Jacob et al., 2021). As the performance of biodiesel is largely affected by microalgal fatty acid desaturation with a preference for saturated fatty acids (SFAs) and monounsaturated fatty acids (MUFAs) (Deshmukh et al., 2019), research interests have been directed toward the modification of fatty acid desaturation. Additionally, lipid with much oleic acid and little linolenic acid is preferable for cooking, since it is more stable and has a longer shelf life (Ton et al., 2020). Nitrogen deprivation is the most common and effective means of inducing a variation in the fatty acid profile, triggering the accumulation of SFAs and MUFAs (Yaakob et al., 2021). However, the detailed scenario and underlying mechanism require further study.

Lipidomics provides a method of deciphering detailed changes in individual lipid species with diverse desaturation. Generally, the accumulation of triacylglycerol (TAG) is accompanied by reduced phospholipids and glycolipids after nitrogen starvation. In *Scenedesmus* sp., TAG dramatically increased with the decrease in monogalactosyldiacylglycerol (MGDG), phosphatidylglycerol (PG), diacylglycerol-N,N,N-trimethylhomoserine (DGTs), and phosphatidylethanolamine (PE), while digalactosyldiacylglycerol (DGDG) and lyso-DGTS first increased and then decreased (Wang et al., 2019). With respect to the fatty acid moieties, polyunsaturated fatty acid (PUFA) decreased in DGDGs and sulfoquinovosyldiacylglycerols (SQDGs), MUFA increased in SQDGs, while all PGs decreased in nitrogen-depleted *Ettlia oleoabundans* (Matich et al., 2018). After 4 h of nitrogen starvation, 16:0, 18:1, 18:2n6, 18:3n3, and 18:3n6 contributed to the accumulation of TAG in *Chlamydomonas reinhardtii*; the proportion of 16:0, 18:1, and 18:2n6 in DGDG and DGTS obviously increased; while some MGDG, DGDG, and DGTS species with PUFA decreased (Yang et al., 2018). Polar lipidomic profiling of nitrogen-absent *Chlorella* sp. showed that acyl moieties of polar lipids shifted towards more saturate, in which the reduction in MGDG (18:3/16:4), DGDG (18:3/16:4), SQDG (18:3/16:0) and highly unsaturated phosphatidylcholine (PC), DGTs, and PG mainly contributed (White et al., 2019). However, there is limited intact and dynamic lipidomics for *Chlorella* sp., whose lysolipids are important for variations in fatty acid desaturation as well.

Multiple transcriptomic analyses were conducted to reveal the underlying regulatory mechanisms determining microalgal fatty acid desaturation, in which the fatty acid desaturase (FAD) pathway was first examined. Indeed, the increased 18:1 content was closely related to upregulated Δ9 fatty acid desaturase in nitrogen-depleted *Auxenochlorella pyrenoidosa* (Zhang et al., 2018). Similarly, the decrease in PUFA (18:3 and 16:3) and an increase of SFA (18:0 and 16:0) and MUFA (18:1 and 16:1) were supported by the downregulation of Δ12-FAD and Δ15-FAD in *Ettlia oleoabundans* (Sturme et al., 2018). In addition, the substrate preference of thioesterase (Chen et al., 2017), fatty acid exporter (Li et al., 2019), lysophosphatidic acid acyltransferase (LPAAT) (Kim et al., 2018), and diacylglycerol acyltransferases (DGATs) (Xin et al., 2019) also contribute to fatty acid desaturation. All of these enzymes were involved in *de novo* fatty acid synthesis or TAG synthesis. Moreover, lipolysis releasing fatty acids and head groups catalyzed by lipases and the β-oxidation pathway degrading fatty acids to acetyl-CoA constitute lipid catabolism (Kong et al., 2018), whose role in fatty acid composition and lipid content is gaining more and more attention. Phospholipid: diacylglycerol acyltransferase (PDAT) from *Saccharomyces cerevisiae* utilized a wide range of phospholipid substrates and preferred 18:0/18:1 acyl donor (Feng et al., 2019). The mutation of MGDG-specific lipase PLASTID GALACTOGLYCEROLIPID DEGRADATION1 (PGD1) in *C. reinhardtii* led to a reduced level of 16:2, 16:3, and 18:2 but higher levels of 16:1, 16:4, and C18:3 (Du et al., 2018). Therefore, it is important to explore the contributions of individual enzymes and different pathways during dynamic periods of nitrogen deprivation.

Many transcription factors have been reported to participate in lipid metabolic pathways. NobZIP77 downregulated the expression of a type-2 diacylgycerol acyltransferase under nitrogen repletion in *Nannochloropsis oceanica* (Zhang et al., 2022). CzMYB1 was correlated with many genes involved in the *de novo* fatty acid synthesis, fatty acid activation and desaturation, membrane lipid turnover, and TAG assembly (Shi et al., 2022). NRR1, a SQUAMOSA promoter-binding protein (SBP), was upregulated only under nitrogen stress, and TAG accumulation decreased by 50% after its mutation (Boyle et al., 2012). Current research focuses on regulating TAG synthesis by transcription factors; however, which transcription factors participate in regulating fatty acid desaturation under nitrogen deprivation and how much each contributes must still be explored and verified.

In this study, we demonstrated the dynamic changes in growth, biochemical composition, and fatty acid desaturation of *Chlorella pyrenoidosa* under nitrogen-deprived conditions. Variations in glycerolipid species were further determined by lipidomics, emphasizing the contribution of lipid turnover at the early stage of nitrogen deprivation. Transcriptomics further affirmed the role of lipid turnover and identified many differentially expressed transcription factors. Finally, two SBPs were selected to be overexpressed in *Yarrowia lipolytica*, demonstrating their contributions to variations in fatty acid desaturation. This study contributes to a better understanding of the molecular mechanisms of variations in algal fatty acid desaturation and will contribute to future improvements in genetic engineering, helping improve the traits of microalgae.

## Materials and methods

### Microalgae strain and growth conditions

*Chlorella pyrenoidosa* FACHB-10 was obtained from Freshwater Algae Culture Collection at the Institute of Hydrobiology. *Chlorella pyrenoidosa* was grown in a 1-L Erlenmeyer flask (200 mm length, 100 mm diameter) with 600 ml working volume of modified BG-11 medium at 25 ± 1°C and 6,000 lx. BG-11 medium consisted of (per liter) 1.5 g NaNO_3_, 0.04 g K_2_HPO_4_·3H_2_O, 0.075 g MgSO_4_·7H_2_O, 0.036 g CaCl_2_·2H_2_O, 0.006 g citric acid, 0.006 g ferric ammonium citrate, 0.001 g EDTA·2Na, 0.02 g Na_2_CO_3_, and 1 ml micronutrient solution. The micronutrient solution consisted of (per liter) 2.86 g H_3_BO_3_, 1.81 g MnCl_2_·4H_2_O, 0.222 g ZnSO_4_·7H_2_O, 0.39 g NaMoO_4_·2H_2_O, 0.079 g CuSO_4_·5H_2_O, and 0.0494 g Co(NO_3_)_2_·6H_2_O. Nitrogen repletion (1.5 g L^−1^ nitrate) was set as control, while the initial nitrate concentration was 0 g L^−1^ for nitrogen deprivation.

### Quantification of biochemical cell composition

Total lipids were extracted from *C. pyrenoidosa* cells with modifications to our previous method (You et al., 2019). A 2-ml solvent mixture of chloroform:methanol (2:1, v/v) was used to suspend freeze-dried algae power (0.1 g). After homogenizing for 5 min with a grinding machine (Jingxin, Shanghai), the samples were centrifuged at 12,000 rpm for 5 min (Eppendorf, Germany). The operation was repeated five times until all lipids were fully recovered. Then, the solvent phase from each replicate was combined and evaporated at 60°C. An analytical balance (Sartorius, Germany) was used to weigh the total lipids.

To determine carbohydrate content, freeze-dried algae power (0.02 g) was suspended in 4 ml of 6 M HCl and 6 ml of distilled water and boiled for 30 min until the carbohydrate was hydrolyzed into monosaccharide, which was detected by an iodine solution. The samples were then determined using the 3.5-dinitrosalicylic acid colorimetric method, according to the manufacturer’s instructions (TC0029, LEAGENE, Beijing).

The colorimetric approach was used to determine the protein concentration. Freeze-dried algae power (0.02 g) was suspended in 1 ml of buffer containing 2% (w/v) sodium dodecyl sulfate (SDS), 10% (v/v) glycerol, 50 mM Tris–HCl (pH 8), and 1 mM dithiothreitol (DTT). The samples were homogenized and centrifuged at 12,000 rpm for 20 min. The BCA kit (PA115, Tiangen, Beijing) was used to determine the protein level of the supernatant fraction.

### Fatty acid methyl ester profile analysis

After acidic transesterification of lipid for 2 h (Zhang et al., 2018), the composition of fatty acid methyl ester (FAME) was investigated. The sample was mixed with 1 ml hexane and 1 ml sodium chloride water solution, then vibrated gently and centrifuged. One microliter of the hexane phase was injected into a 7890B/5977B gas chromatography–mass spectrometry (Agilent, Germany) equipped with HP-5MS (5% phenyl)-methylpolysiloxane nonpolar capillary column (30 m × 0.25 mm × 0.25 μm). Inlet temperature was 270°C, and the column temperature was kept at 140°C for 1 min and then increased to 220°C with a temperature gradient of 4°C min^−1^, maintained for 4 min, finally reached 300°C with a temperature gradient of 30°C min^−1^, and maintained for 2 min. Helium as carrier gas was set to a column flowrate of 1 ml min^−1^. The mass spectrometry was performed in positive electron ionization mode at 70 eV, obtaining full scan spectra with an m/z range of 45−550. The FAME components were identified by matching their electron ionization mass spectra with those from the NIST 17 MS library. For quantification, nonadecanoic acid was added as an internal standard.

### Lipidomic analysis by ultra-high performance liquid chromatography–mass spectrometry

Total lipids were extracted from *C. pyrenoidosa* cells under nitrogen repletion and deprivation on days 2, 6, and 12. Isopropanol:methanol (1:1, v/v) was used to redissolve lipids to a final concentration of 1 mg ml^−1^. Lipid substances were separated by a Vanquish UHPLC system equipped with an ACQUITY UPLC BEH C18 column (100 × 2.1 mm, 1.7 μm, Waters), and mass spectrometry was performed on a Thermo Scientific Q Exactive hybrid quadrupole-Orbitrap mass spectrometer with an electrospray ionization source, using a previously reported method (Peng et al., 2019).

Using the XCalibur software (Thermo Fisher Scientific), the data were extracted as raw files. LipidSearch 4.1 (Thermo Fisher Scientific, USA) was used to perform identification, alignment, and quantification. By comparing the fragmentation ions, retention time, and high-resolution mass using the LipidSearch Database, the lipids were identified, which revealed the fatty acyl groups (FAs) on lipids. The sample names (based on the retention time, peak feature, and m/z) and response areas were exported for further analyses. LINT-web (http://www.lintwebomics.info/) was used for statistical analyses and lipidomic data mining (Li et al., 2021), and LION-web (www.lipidontology.com) was used for ontology enrichment analyses (Molenaar et al., 2019).

### RNA extraction, sequencing, and transcriptome analysis

Total RNA was isolated from microalgae cells with Plant RNA Purification Reagent (Invitrogen, USA) according to the manufacturer’s instructions after 2 days of cultivation. The quality of the RNA was then evaluated using a 2100 Bioanalyzer (Agilent, Germany) and quantified by the NanoDrop ND-2000 spectrophotometer (NanoDrop Technologies, USA). RNA sequencing and data analysis were performed by Majorbio (Shanghai Majorbio BioPharm Technology). Relevant RNA sequencing raw data were deposited in the GenBank repository under accession number PRJNA855982. The *p*-value obtained from the statistical test was corrected using the false discovery rate (FDR) adjustment. Differentially expressed genes (DEGs) were identified according to |log_2_FC| ≥ 1 and *p*-adjust < 0.05. The Kyoto Encyclopedia of Genes and Genomes (KEGG) database (http://www.genome.jp/kegg/) was used to annotate and analyze the metabolic pathways.

### Relative gene expression level detected by RT-qPCR

The FastKing RT Kit (With gDNase) (Tiangen, Beijing) was used to synthesize cDNA. All PCR primers used in this study are listed in **Supplementary Table S1**. Real-time PCR was performed on qTOWER3G touch (Analytikjena, Germany) using SuperReal PreMix Plus (SYBR Green) (Tiangen, Beijing). The reactions began at 95°C for 15 min, then 40 cycles of 95°C for 10 s and 60°C for 30 s, followed by a melting curve step at 60°C–95°C. Three biological replicates were used for each reverse transcription quantitative PCR (RT-qPCR) assay. The relative expression levels were normalized to the β-actin gene and were calculated using the 2^−ΔΔCT^ method (Livak and Schmittgen, 2001).

### Construction and cultivation of the *Yarrowia lipolytica* transformants with heterologously expressed SBP

*Yarrowia lipolytica* CLIB122 (Δura3 Δhis Δleu2) and plasmid pINA1313 were kindly provided by Professor Hairong Cheng (Shanghai Jiao Tong University). *SBP1* and *SBP2* were PCR amplified using the primers homo-SBP1-F/R and homo-SBP2-F/R listed in **Supplementary Table S1** and subcloned to the plasmid pINA1313 with the Hieff One Step Cloning Kit (Yeasen, Shanghai). The pINA1313-SBP plasmid was linearized and transformed into *Y. lipolytica* CLIB122 according to previously published methods (Li et al., 2016). To select the transformants, cells were spread on the YNG minimal medium and incubated at 30°C for 3–5 days until colonies appeared. The correct transformants with *SBP1* or *SBP2* heterologously expressed were verified by genome PCR and RT-qPCR using the primers listed in **Supplementary Table S1**. The YNG minimal medium contained, per liter, 6.7 g of yeast nitrogen base (without amino acids), 5.0 g of ammonia sulfate, 20.0 g of glucose, 0.02 g of leucine, and 0.02 g of histidine. For growth and fatty acid composition analysis, the HGM medium contained, per liter, 6.7 g of yeast nitrogen base (without amino acids), 3.0 g of ammonia sulfate, 1.0 g of yeast extract, 1.5 g of MgSO_4_·7H_2_O, 0.0015 g thiamine hydrochloride, 80 g glucose, 6.3 g KH_2_PO_4_, 2.7 g K_2_HPO_4_, 0.02 g leucine, and 0.02 g histidine.

### Statistical analysis

Three biological replicates were performed for all experiments, with results presented as mean ± standard deviation (SD). Statistical analysis was performed using GraphPad Prism 9 (GraphPad, USA). The *p*-values were calculated *via* Student’s *t-*test.

## Results

### Growth and dynamic changes of biochemical composition under nitrogen deprivation

**Figure 1:**
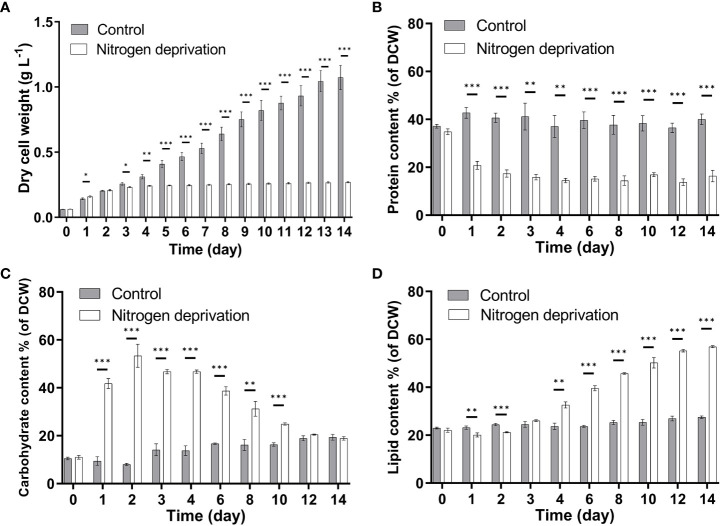
Effects of nitrogen deprivation on growth and biochemical composition of *Chlorella pyrenoidosa*. **(A)** Biomass yield. **(B)** Protein content. **(C)** Carbohydrate content. **(D)** Lipid content. DCW, dry cell weight. Values are mean ± SD (n = 3). Student’s *t-*test was performed to determine statistically significant differences: *p < 0.05, **p < 0.01, and ***p < 0.001.

Nitrogen is a macro-element used to synthesize nuclear acids, proteins, cell walls, and membranes, whose absence will cause severe changes in growth and biochemical composition. Under nitrogen deprivation, *C. pyrenoidosa* stopped growing on the second day, and the dry cell weight largely remained stable, eventually reaching 0.27 g L^−1^. This was only 23.48% of that under nitrogen repletion (**Figure 1A**). Therefore, nitrogen deficiency could significantly inhibit the growth of *C. pyrenoidosa*. In addition, when nitrogen was depleted, the protein content rapidly decreased to 20.84% on the second day (**Figure 1B**), while the carbohydrate content dramatically increased to 53.37% (**Figure 1C**). This indicates that *C. pyrenoidosa* could degrade its protein and accumulate carbohydrate during the early stages of nitrogen deprivation. As nitrogen deprivation continued, the content of carbohydrate gradually decreased and eventually accounted for 18.88% of dry cell weight (**Figure 1C**), while the lipid content gradually increased (**Figure 1D**), indicating that there could be carbohydrate-to-lipid conversions. On the 14th day, lipid content accounted for 56.90% of dry cell weight, which was 2.07-fold of that under nitrogen repletion (**Figure 1D**). Altogether, nitrogen deprivation caused the arrest of growth, accumulation of carbohydrate under early nitrogen deprivation, and lipid accumulation subsequently.

### Variations in the fatty acid profile under nitrogen deprivation

Nitrogen deprivation also triggers severe variation in the microalgal fatty acid profile. Under nitrogen repletion, polyunsaturated fatty acids such as 16:3, 18:2, and 18:3 were the main components, while 18:1 accounted for only 1.7%–3.7% of total fatty acids (**Table 1**). However, when nitrogen was depleted, the content of 18:1 increased rapidly, reaching 4.88%, 9.97%, and 11.90% on the 2nd, 6th, and 12th days, which were 2.87-, 3.69-, and 3.22-fold of that under nitrogen repletion, respectively (**Table 1**). Meanwhile, the content of 18:3 decreased from 19.46% to 25.89% to 12.14%–16.05%, while 18:0 increased from 4.6%–7.61% to 6.84%–13.62%. In addition, the proportion of 16:1, 16:2, and 16:3 continuously decreased while 16:0 increased to 48.45% on the second day and then gradually decreased. The content of polyunsaturated fatty acids decreased from 55.43%–62.43% to 33.48%–40.34%, saturated fatty acids increased from 28.04%–39.35% to 46.77%–56.64%, and monounsaturated fatty acids increased from 2.70%–8.87% to 6.12%–12.27% under nitrogen-deficient conditions. These results indicate that nitrogen deprivation significantly reduced fatty acid desaturation in *C. pyrenoidosa*.

**Table 1:** Dynamic variation of fatty acid profile in *Chlorella pyrenoidosa* under nitrogen repletion and deprivation conditions.

Fatty acid (%)	Day 2			Day 6		Day 12	
	CON	ND		CON	ND	CON	ND
C14:0	0.30 ± 0.01	0.28 ± 0.04		0.41 ± 0.01	0.47 ± 0.04	0.42 ± 0.04	0.46 ± 0.07
C15:0	nd	0.19 ± 0.14		nd	0.17 ± 0.04**	nd	0.17 ± 0.02***
C16:0	22.67 ± 0.29	48.45 ± 0.67***		29.70 ± 1.63	40.65 ± 1.56**	30.86 ± 1.35	35.89 ± 1.20*
C16:1	7.16 ± 0.08	1.25 ± 0.15***		nd	0.24 ± 0.17	0.62 ± 0.88	0.37 ± 0.03
C16:2	7.48 ± 0.13	1.53 ± 0.13***		3.01 ± 0.70	0.79 ± 0.08*	1.56 ± 0.15	0.76 ± 0.07**
C16:3	18.59 ± 0.20	10.48 ± 0.74***		14.80 ± 0.93	6.78 ± 0.36***	14.07 ± 0.68	6.73 ± 0.53***
C17:0	0.48 ± 0.05	0.89 ± 0.08**		0.53 ± 0.02	0.66 ± 0.04*	0.47 ± 0.02	0.61 ± 0.02**
C18:0	4.60 ± 0.26	6.84 ± 0.38**		8.05 ± 0.49	13.62 ± 0.73***	7.61 ± 1.18	9.64 ± 2.55
C18:1	1.70 ± 0.01	4.88 ± 0.33***		2.70 ± 0.51	9.97 ± 0.43***	3.70 ± 0.18	11.90 ± 1.55**
C18:2	16.90 ± 0.16	12.73 ± 0.19***		16.73 ± 0.24	13.10 ± 0.52***	13.90 ± 0.50	16.80 ± 1.05*
C18:3	19.46 ± 0.31	12.14 ± 0.32***		23.17 ± 0.36	12.82 ± 0.75***	25.89 ± 0.09	16.05 ± 0.75***
C20-C26	0.66 ± 0.04	0.36 ± 0.10*		0.90 ± 0.06	0.74 ± 0.06	0.90 ± 0.05	0.62 ± 0.20
SFA	28.04 ± 0.51	56.64 ± 0.97***		38.69 ± 1.76	55.56 ± 2.17**	39.35 ± 2.19	46.77 ± 3.73
UFA	71.30 ± 0.48	43.00 ± 1.06***		60.41 ± 1.72	43.70 ± 2.21**	59.75 ± 2.24	52.62 ± 3.85
PUFA	62.43 ± 0.54	36.88 ± 1.19***		57.71 ± 2.19	33.48 ± 1.69***	55.43 ± 1.32	40.34 ± 2.30**
MUFA	8.87 ± 0.08	6.12 ± 0.18***		2.70 ± 0.51	10.22 ± 0.59***	4.32 ± 1.05	12.27 ± 1.58**

Cells under nitrogen repletion (control, CON) and nitrogen deprivation (ND) conditions were harvested on days 2, 6, and 12. Values are mean ± SD (n = 3). Student’s *t*-test was performed to determine statistically significant differences: *p < 0.05, **p < 0.01, and ***p < 0.001.

### Dynamic lipidomic profiling upon nitrogen deprivation

**Figure 2:**
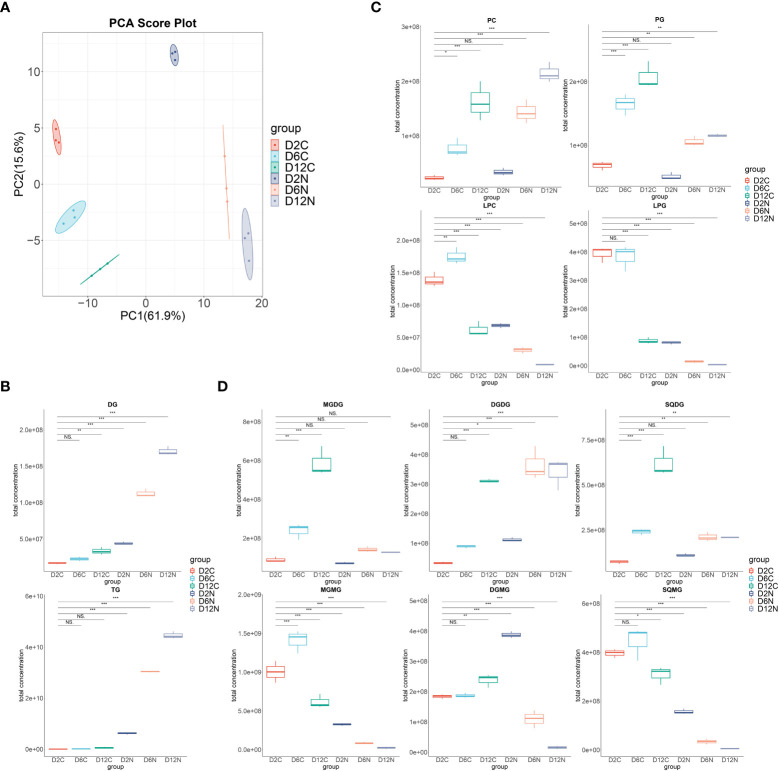
Dynamic changes of the individual lipid classes in *Chlorella pyrenoidosa* under nitrogen repletion and deprivation conditions. **(A)** PCA score plot of six lipidomic datasets. Cells under control (C) and nitrogen deprivation (N) were harvested on days 2, 6, and 12. **(B)** The box–whisker plots of main neutral lipids. DG, diacylglycerol; TG, triacylglycerol. **(C)** The box–whisker plots of main phospholipids. PC, phosphatidylcholine; PG, phosphatidylglycerol; LPC, lysophosphatidylcholine; LPG, lysophosphatidylglycerol. **(D)** The box–whisker plots of main glycolipids. MGDG, monogalactosyldiacylglycerol; DGDG, digalactosyldiacylglycerol; SQDG, sulfoquinovosyldiacylglycerol; MGMG, monogalactosylmonoacylglycerol; DGMG, digalactosylmonoacylglycerol; SQMG, sulfoquinovosylmonoacylglycerols. Lipid classes whose total concentration exceeded 1.0e+08 were presented; all lipid classes are shown in **Supplementary Figure S2**. Values are mean ± SD (n = 3). Student’s *t-*test was performed to determine statistically significant differences: NS, no significance, *p < 0.05, **p < 0.01, and ***p < 0.001.

To assess the individual lipid species that comprise the total fatty acids profile, dynamic glycerolipid changes in *C. pyrenoidosa* under control and nitrogen deprivation were analyzed by ultra-high performance liquid chromatography–tandem mass spectrometry (UHPLC-MS/MS)-based lipidomics. Principal component analysis (PCA) was employed to perform dimensionality reduction analyses on the dataset. The first two components explained 77.5% of the variance (**Figure 2A**). In the score plot, lipids of cells growing under nitrogen repletion were clearly separated from those of nitrogen-deprived cells.

In **Figures 2B–D**, the successively increased content of diacylglycerols, triacylglycerols, phospholipids, and glycolipids were accompanied by the decrease in all lysophospholipids and lysoglycolipids upon nitrogen deprivation. Consistently, in the LION enrichment analysis (**Supplementary Figure S1**), the most significant LION terms were triacylglycerol, lipid storage, and lipid droplet on the 2nd, 6th, and 12th days, highlighting the dominant status of TAG in lipid species alteration. The most significant two fatty acids were 16:0 followed by 18:1 on the second day, while they were first 18:1 then 16:0 on the 6th and 12th days, respectively, consisting with the variations in the fatty acid profile under nitrogen deprivation conditions.

**Figure 3:**
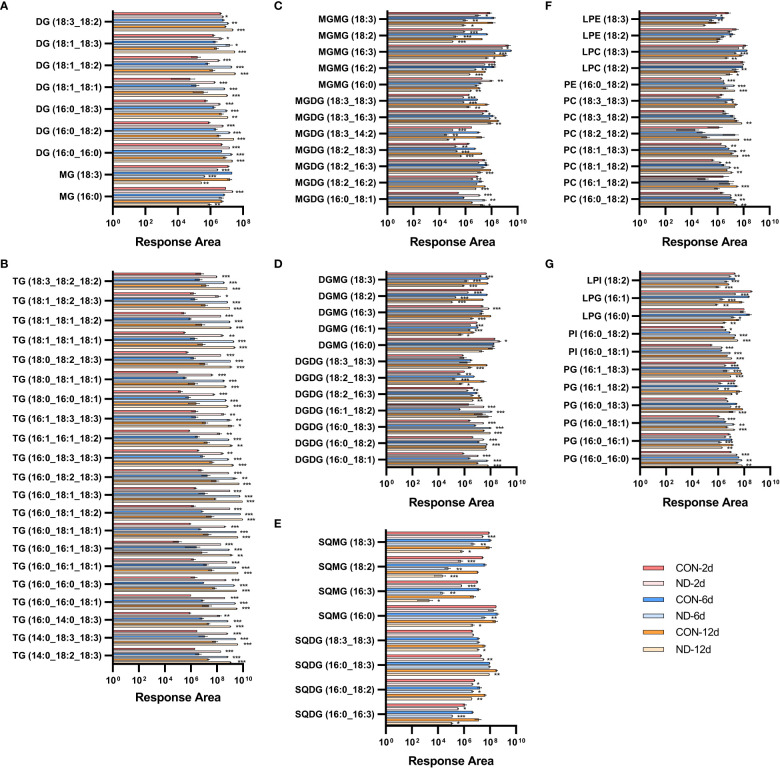
Dynamic changes in individual lipid species in *Chlorella pyrenoidosa* under nitrogen repletion and deprivation conditions. **(A, B)** Response area of neutral lipids. MG, monoacylglycerol; DG, diacylglycerol; TG, triacylglycerol. **(C–E)** Response area of glycolipids. MGDG, monogalactosyldiacylglycerol; DGDG, digalactosyldiacylglycerol; SQDG, sulfoquinovosyldiacylglycerol; MGMG, monogalactosylmonoacylglycerol; DGMG, digalactosylmonoacylglycerol; SQMG, sulfoquinovosylmonoacylglycerols. **(F, G)** Response area of phospholipids. PC, phosphatidylcholine; PE, phosphatidylethanolamine; PG, phosphatidylglycerol; PI, phosphatidylinositol; LPC, lysophosphatidylcholine; LPE, lysophosphatidylethanolamine; LPG, lysophosphatidylglycerol; LPI, lysophosphatidylinositol. Triacylglycerol species whose response area was more than 5.0e+08, and other lipid species with more than 1.0e+07 were presented; all lipid species are shown in **Supplementary Data 1**. Values are mean ± SD (n = 3). Student’s *t-*test was performed to determine statistically significant differences: *p < 0.05, **p < 0.01 and ***p < 0.001.

To reveal the contribution of the Kennedy pathway and membrane lipid turnover in triacylglycerol accumulation and fatty acid desaturation variation, dynamic changes in all lipid species during nitrogen deprivation were further investigated (**Figure 3**; **Supplementary Data 1**).

#### Variation of neutral lipids

Triacylglycerols are the main form of neutral lipids in microalgae. All of these species exhibited rapid and sustained increases on the 2nd, 6th, and 12th days of nitrogen deprivation (**Figure 3B**). TAG species containing 16:0 or 18:1 were the main components, i.e., 16:0_16:0_18:1/18:3, 16:0_16:1_18:1/18:3, 16:0_18:1_18:1/18:2/18:3, 16:0_18:2/18:3_18:3, 16:1_16:1_18:2, 16:1_18:3_18:3, 18:0_18:2_18:3, 18:1_18:1_18:1/18:2, and 18:1_18:2_18:3, while there was less for those with 16:2 or 16:3. The diacylglycerol (DAG) species, namely,16:0/18:1/18:2/18:3_16:0 (prokaryotic pathway) and 18:1/18:2/18:3_18:1 (eukaryotic pathway), significantly increased in line with the elevated generation of TAG (**Figure 3A**), providing precursors for their assembly. The C16-DAG pool increased remarkably and became the main form of DAG on the second day, while C18-DAG was gradually outstripped with continued nitrogen stress. In addition, the content of 16:0-monoacylglycerol (MAG) significantly increased on the second day and then decreased (**Figure 3A**), confirming the predominant role of lipids assembled by prokaryotic pathways under early nitrogen deprivation while eukaryotic pathway in subsequent periods.

#### Variation of phospholipids and lysophospholipids

Phospholipids can supply the acyl moiety for TAG assembly by PDAT or be broken down to DAG, fatty acids, and glycerol-3-phosphate by phospholipases, providing precursors of TAG and galactolipids (Xu et al., 2018). In *C. pyrenoidosa*, the major variant phospholipids species were PC, PG, lysophosphatidylcholine (LPC), and lysophosphatidylglycerol (LPG). The PC species, namely, 18:1/18:2_16:0 (prokaryotic pathway), 18:1/18:2/18:3_18:0, and 18:1_18:1/18:2/18:3 (eukaryotic pathway), increased significantly, while other species decreased on the second day (**Figure 3F**; **Supplementary Data 1**). Accordingly, in LPC species, 16:0 and 18:3 decreased while 18:1 and 18:2 exhibited no difference on the second day, then all species decreased except 16:0. PDAT transfers the acyl group from sn-2 of PC to DAG generating TAG (Xu et al., 2018), causing the early decrease in 16:0-LPC. All main PG species decreased significantly, except 16:0_16:0 and 16:0_18:1, with almost all LPG species significantly decreasing in addition to 18:1-LPG (**Figure 3G**). The situation was similar in PE, phosphatidylinositol (PI), phosphatidylserine (PS), and corresponding lysophospholipids. Phospholipids containing 16:0 or 18:1 seemed to replace others to maintain the level of phospholipids, which progressively increased, while lysophospholipids constantly decreased.

#### Variation of galactolipids and lysogalactolipids

Galactolipases, such as PGD1 and heat-inducible lipase1 (HIL1), could remove C18:1 and C18:3, respectively, from MGDG (Yu et al., 2021). Under nitrogen deprivation, MGDG species with 16:0 or 18:1 significantly accumulated, i.e., 16:0/18:0/18:1/18:2/18:3_16:0, while those with non-16/18 carbon and more unsaturated species decreased. These changes were similar in DGDG and SQDG (**Figures 3C-E**; **Supplementary Data 1**). In monogalactosylmonoacylglycerol (MGMG) species, 16:0 increased on the 2nd day and then decreased on the 6th and 12th days, while 16:2, 16:3, 18:2, and 18:3 decreased to varying degrees. The changes were similar in digalactosylmonoacylglycerol (DGMG) in addition to the increased 16:3, while all sulfoquinovosylmonoacylglycerols (SQMG) species decreased. It seemed that 18:1/18:3_16:0-MGDG and DGDG were hydrolyzed by galactolipases, causing the accumulation of 16:0-MGMG and 16:0-DGMG under early nitrogen deprivation.

Collectively, these results illustrate changes in lipid species with different fatty acid chain lengths and desaturation, supporting the crucial role of lipid turnover in TAG accumulation and variation of fatty acid desaturation.

### Global transcriptional characteristics in response to nitrogen deprivation

Most transcriptomic analyses in nitrogen-deprived microalgae focus on triacylglycerol synthesis and regard fatty acid desaturase as the main contributor to fatty acid desaturation (Sturme et al., 2018; Sun et al., 2019). In this study, the transcriptome was conducted on the second day to identify the contribution of FADs and other enzymes associated with lipid metabolism, based on earliest experimental variations in the fatty acid profile and lipid species. The correlation between samples was good (**Supplementary Figure 3A**), and 8,460 genes were significantly differentially expressed under nitrogen deprivation condition. Of these, 3,671 genes were upregulated, and 4,789 genes were downregulated (**Supplementary Figure 3B**). Gene Ontology (GO) annotation analysis showed that nitrogen deprivation had the most significant effect on metabolic and cell processes and membrane and cell parts (**Supplementary Figure 3C**). KEGG functional enrichment analysis revealed that differentially expressed genes were mainly enriched in pathways related to genetic information processing and some fatty acid metabolism, such as biosynthesis of unsaturated fatty acids, α-linolenic acid metabolism, and fatty acid degradation (**Supplementary Figure S3D**).

To validate the expression level, 13 differentially expressed genes were tested by RT-qPCR, of which 12 genes (92.86%) were differentially expressed consistently with transcriptome sequencing results (**Supplementary Table S2**), confirming the reliability of transcriptome data.

#### Nitrogen metabolism and photosynthesis

Key enzymes in the nitrogen assimilation pathway, such as nitrate transporter (Nrt), nitrate reductase (NR), and glutamine synthetase (GS), were upregulated (**Supplementary Data 2**), indicating increased assimilation of nitrogen in response to nitrogen deprivation. Photosynthesis is essential to fix inorganic carbon to organic utilizing light energy for microalgae. Almost all genes of the light-harvesting complex (LHC) were downregulated. The expression of nine genes were lower, while only two were more highly expressed in photosystem I (PS I) and photosystem II (PS II), indicating degenerative photosynthesis. Enzymes in the Calvin cycle, such as ribulose-1,5-bisphosphate carboxylase/oxygenase (Rubisco), phosphoglycerate kinase (PGK), and glyceraldehyde 3-phosphate dehydrogenase (GAPDH) were significantly upregulated, which meant that carbon fixation was ongoing to consume excess photosynthetic energy.

#### Starch and protein metabolism

**Figure 4:**
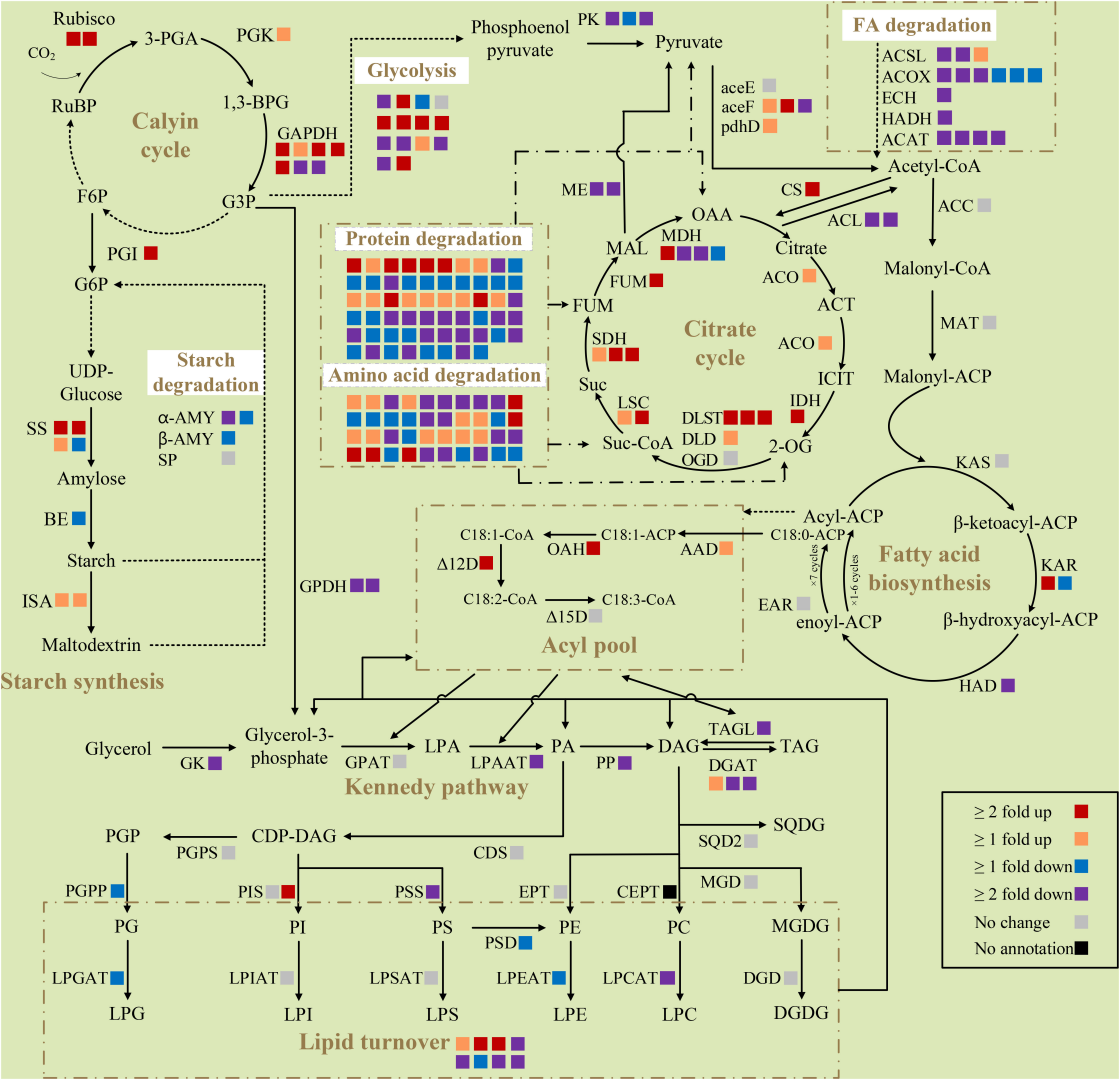
Differentially expressed genes of selected pathways respond to nitrogen deprivation in *Chlorella pyrenoidosa*. Each square box indicates a gene isoform annotated in the transcriptome. Different colors of boxes represent the differential expression of genes, and the explanation is shown at the bottom right of the figure. All presented fold changes are statistically significant, *p* < 0.05. Reactions whose enzymes are not shown are denoted as dashed arrows. RuBP, ribulose 1,5-bisphosphate; 3-PGA, 3-phosphoglyceric acid; 1,3-BPG, 1,3-biphosphoglycerate; F6P, fructose 6-phosphate; G3P, glyceraldehyde-3-phosphate; G6P, glucose-6-phosphate; OAA, oxaloacetic acid; ACT, aconitate; ICIT, isocitrate; 2-OG, α-ketoglutarate; Suc-CoA, succinyl-CoA; Suc, succinic acid; FUM, fumarate; MAL, malic acid; ACP, acyl carrier protein; LPA, lysophosphatidic acid; PA, phosphatidic acid; DAG, diacylglycerol; TAG, triacylglycerol; CDP, cytidine diphosphate; PGP, phosphatidylglycerolphosphate; PC, phosphatidylcholine; PE, phosphatidylethanolamine; PG, phosphatidylglycerol; PI, phosphatidylinositol; PS, phosphatidylserine; LPC, lysophosphatidylcholine; LPE, lysophosphatidylethanolamine; LPG, lysophosphatidylglycerol; LPI, lysophosphatidylinositol; LPS, lysophosphatidylserine; MGDG, monogalactosyldiacylglycerol; DGDG, digalactosyldiacylglycerol; SQDG, sulfoquinovosyldiacylglycerol; Rubisco, ribulose diphosphate carboxylase; PGK, phosphoglycerate kinase; GAPDH, glyceraldehyde 3-phosphate dehydrogenase; PGI, phosphoglucose isomerase; SS, starch synthase; BE, 1,4-a-glucan branching enzyme; α-AMY, α-amylase; β-AMY, β-amylase; SP, starch phosphorylase; ISA, isoamylase; PK, pyruvate kinase; aceE, pyruvate dehydrogenase E1 component; aceF, pyruvate dehydrogenase E2 component; pdhD, dihydrolipoamide dehydrogenase; ACSL, long chain acyl-CoA synthase; ACOX1, acyl-CoA oxidase; ECH, enoly-CoA hydratase; HADH, 3-hydroxyacyl-CoA dehydrogenase; ACAT, acetyl-CoA C-acetyltransferase CS, citrate synthase; ACL, ATP-citrate synthase; ACO, aconitate hydratase; IDH, isocitrate dehydrogenase; DLST, dihydrolipoamide succinyltransferase; DLD, dihydrolipoyl dehydrogenase; OGD, 2-oxoglutarate dehydrogenase; LSC, succinyl-CoA synthetase; SDH, succinate dehydrogenase; FUM, fumarate hydratase; MDH, malate dehydrogenase; ME, malic enzyme; ACC, acetyl-CoA carboxylase; MAT, malonyl-CoA ACP transacylase; KAS, beta-ketoacyl-ACP synthase; KAR, beta-ketoacyl-ACP reductase; HAD, beta-hydroxyacyl-ACP dehydrase; EAR, enoyl-ACP reductase; AAD, acyl-ACP desaturase; OAH, oleoyl-ACP hydrolase; Δ12D, Δ12-desaturase; Δ15D, Δ15-desaturase; GK, glycerol kinase; GPDH, glycerol-3-phosphate dehydrogenase; GPAT, glycerol-3-phosphate O-acyltransferase; LPAAT, lysophosphatidic acid acyltransferase; PP, phosphatidate phosphatase; DGAT, diacylglycerol O-acyltransferase; TAGL, triacylglycerol lipase; CDS, CDP-diglyceride synthase; PGPS, phosphatidylglycerolphosphate synthase; PGPP, phosphatidylglycerolphosphate phosphatase; PIS, phosphatidylinositol synthase; PSS, phosphatidylserine synthase; PSD, phosphatidylserine decarboxylase; EPT, diacylglycerol ethanolaminephosphotransferase; CEPT, diacylglycerol cholinephosphotransferase; LPGAT, lyosphosphatidylglycerol acetyltransferase; LPCAT, lysophosphatidylcholine acyltransferase; LPEAT, lyosphosphatidylethanolamine acyltransferase; LPSAT, lyosphosphatidylserine acyltransferase; LPIAT, lyosphosphatidylinositol acyltransferase; MGD, 1,2-diacylglycerol 3-beta-galactosyltransferase; DGD, digalactosyldiacylglycerol synthase; SQD2, sulfoquinovosyltransferase.

Enzymes involved in starch synthesis, especially starch synthase (SS), the key enzyme in the starch synthesis pathway, were upregulated, while α-amylase (α-AMY) and β-amylase (β-AMY) related to its hydrolysis were significantly downregulated (**Figure 4**). This was consistent with the increased carbohydrate content in cells at this time. The protein content of *C. pyrenoidosa* dramatically decreased on the second day of nitrogen deprivation; consistently, many genes encoding protease in proteasome and ubiquitin-mediated proteolysis, such as papain family cysteine protease and E3 ubiquitin-ligase, were upregulated. In addition, many genes involved in amino acid degradation exhibited significant upregulation, indicating carbon flow switching from protein to carbohydrate under early nitrogen deprivation.

#### Fatty acid biosynthesis and acetyl-CoA metabolism

Most genes involved in fatty acid synthesis, including the key enzyme acetyl-CoA carboxylase (ACC), had no significant change, suggesting constant activity of the fatty acid synthesis pathway (**Figure 4**). Stearoyl-ACP desaturase (AAD), which synthesizes oleyl-ACP, and oleyl-ACP thioesterase (OAH), which dissociates 18:1 from the acyl carrier protein, were upregulated to promote the synthesis of more 18:1. The expression of Δ12 fatty acid desaturase (Δ12D) was upregulated, while Δ15 fatty acid desaturase (Δ15D) did not change, which was consistent with increase in the 18:1 content and decrease in the 18:3 content in cells.

Many genes involved in glycolysis were significantly downregulated. Of these, pyruvate kinase (PK), the rate-limiting enzyme for glycolysis, was also downregulated. Glycolysis converts sugar to pyruvate, which subsequently converts to acetyl-CoA as the substrate of fatty acid biosynthesis (Huang et al., 2016). Attenuated glycolysis meant less carbon flow converted into fatty acid biosynthesis. Although two of three subunits of pyruvate dehydrogenase complex (PDC) were upregulated (**Figure 4**), malic enzyme (ME), which catalyzes the formation of pyruvate from malic acid, was downregulated. Acetyl-CoA could also be used as the substrate of the tricarboxylic acid (TCA) cycle. Upregulated citrate synthase (CS) and downregulated ATP-citrate synthase (ACL) drew a large amount of acetyl-CoA into the TCA cycle. Consistently, almost all genes involved in the TCA cycle were upregulated, likely producing substantial amounts of carbon precursors and energy for fatty acid synthesis or lipid remodeling.

#### Glycerolipid metabolism

Although the glycerol kinase (GK), lysophospholipids acid acyltransferase (LPAAT), and phosphatidic acid phosphatase (PP) were all downregulated, key enzymes in triacylglycerol synthesis, diacylglycerol acyltransferase (DGAT), and major lipid droplet protein (MLDP) were upregulated (**Figure 4**). Meanwhile, the triacylglycerol degradation enzyme (SDP1) and all five enzymes in fatty acids beta oxidation pathways were downregulated (**Figure 4**), which facilitated the accumulation of fatty acids and triacylglycerol. The enzyme encoding choline-phosphate cytidylyltransferase 1 (PCYT1) was upregulated, producing more CDP-choline for phosphatidylcholine synthesis. As for the synthesis of phospholipids, phosphatidylinositol synthase (PIS) was upregulated, while phosphatidyl serine synthase (PSS), phosphatidyl-glycerolphosphate phosphatase (PGPP), lyosphosphatidylglycerol acetyltransferase (LPGAT), lysophosphatidylcholine acyltransferase (LPCAT), and lysophosphatidylethanolamine acyltransferase (LPEAT) were downregulated. For glycolipid biosynthesis, 1,2-diacylglycerol 3-beta-galactosyltransferase (MGD), digalactosyldiacylglycerol synthase (DGD), and sulfoquinovosyltransferase (SQD2) exhibited no change. Overexpression of phospholipid glycerol acyltransferase (presumptive PDAT), phospholipase A, patatin-like phospholipase, FATTY ACID EXPORT 6, and other lipases, and the suppression of many unknown lipases, indicated the crucial role of lipid remodeling in fatty acid desaturation variation.

### Heterologous expression of SBP transcription factors in *Yarrowia lipolytica* CLIB122

Transcription factors play an important role in the growth, development, and environmental stress response of microalgae. Under nitrogen deprivation, 60 differentially expressed transcription factors were identified in *C. pyrenoidosa*, including 16 SBPs, 14 MYBs, 9 ZFs, 8 RWP-RKs, 3 bZIPs, 2 AP2s, and 8 other transcription factors (**Supplementary Figure S4**).

Among the 60 differentially expressed transcription factors, SBPs were the most abundant and highly differentially expressed transcription factors, which are related to plant growth and development, flowering, and abiotic stress response (Song et al., 2019). NRR1, a SQUAMOSA promoter binding protein, was upregulated only under nitrogen stress, and TAG accumulation decreased by 50% after its mutation (Boyle et al., 2012). Since TAGs constituted up to 90% of total fatty acids, and 16:0 and 18:1 were the major fatty acids produced in *C. vulgaris* under nitrogen starvation (Breuer et al., 2012), we speculated that SBP family transcription factors played an important role in the variation in TAG content and, therefore, fatty acid desaturation, which we verified in *Y. lipolytica*.

**Figure 5:**
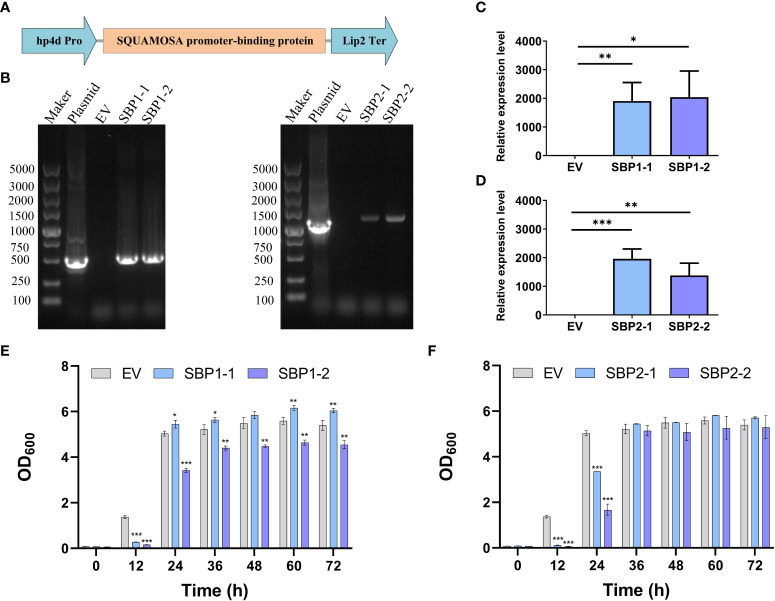
Construction, verification, and growth of *Yarrowia lipolytica* transformants with overexpressed SQUAMOSA promoter-binding protein. **(A)** Diagram of the expression cassette. **(B)** Agarose gel electrophoresis of PCR products with different templates. **(C)** Relative expression level of *SBP1*. **(D)** Relative expression level of *SBP2*. **(E)** Growth of EV and transformants with overexpressed SBP1. **(F)** Growth of EV and transformants with overexpressed SBP2. EV, empty vector; SBP1-1/2, transformants with overexpressed SBP1; SBP2-1/2, transformants with overexpressed SBP2. Values are mean ± SD (n = 3). Student’s *t-*test was performed to determine statistically significant differences: *p < 0.05, **p < 0.01, and ***p < 0.001.

We cloned the intact sequence of *TRINITY_DN3848_c0_g2* and *TRINITY_DN9157_c0_g1*, subsequently known as the *SBP1* and *SBP2*, and inserted them between the hp4d promoter and the Lip2 terminator on the pINA1313-expressing plasmids, respectively (**Figure 5A**). The correct transformants with heterologously expressed *SBP1* or *SBP2* were obtained by transformation, screening, and PCR verification (**Figure 5B**); *SBP1* and *SBP2* were overexpressed in the transformants compared to the control (**Figures 5C, D** **)**. As shown in **Figures 5E, F**, yeast with overexpressed SBP exhibited slower growth and stabilized at 36 h, while the control stabilized at 24 h. There was no clear trend of difference in the final OD_600_.

The fatty acid profiles of the transformants were surveyed, and the content of 18:2 significantly decreased in all transformants (**Table 2**). The content of 18:1 significantly increased in transformants overexpressing SBP1. In transformants overexpressing SBP2, the percentage of 16:1 decreased, while that of 16:0 and 18:0 increased. In terms of fatty acid desaturation, overexpression of SBP1 increased the proportion of MUFA and reduced the proportion of PUFA, while overexpression of SBP2 increased the content of SFA and reduced unsaturated fatty acid (UFA). As for the absolute content, the variation in fatty acid proportion was mainly attributed to the increase of 18:1 and the total fatty acids content (**Table 3**), confirming the function of SBP family transcription factors in promoting fatty acid accumulation and reducing fatty acid desaturation.

**Table 2:** Fatty acid composition of *Yarrowia lipolytica* with overexpressed SQUAMOSA promoter-binding protein.

Fatty acid (%)	EV	SBP1-1	SBP1-2	SBP2-1	SBP2-2
C16:0	15.56 ± 0.16	14.58 ± 0.10**	15.63 ± 0.20	16.21 ± 0.03*	16.63 ± 0.68
C16:1	13.59 ± 0.52	10.98 ± 0.32**	13.74 ± 0.31	11.12 ± 0.07*	11.98 ± 0.08*
C17:1	2.37 ± 0.09	3.08 ± 0.19**	2.39 ± 0.04	3.30 ± 0.10**	2.55 ± 0.20
C18:0	1.89 ± 0.05	2.56 ± 0.17**	2.03 ± 0.09	4.43 ± 0.08***	3.87 ± 0.21***
C18:1	32.76 ± 0.29	38.93 ± 0.25***	37.88 ± 0.53***	36.30 ± 0.09**	33.44 ± 0.86
C18:2	30.80 ± 0.36	26.93 ± 0.06***	26.03 ± 0.42***	25.51 ± 0.05***	27.97 ± 0.74**
Others	3.03 ± 0.15	2.94 ± 0.17	2.30 ± 0.07**	3.11 ± 0.08	3.56 ± 0.06*
SFA	18.67 ± 0.16	18.17 ± 0.19*	18.64 ± 0.18	21.92 ± 0.03***	21.85 ± 0.84**
MUFA	49.57 ± 0.46	53.87 ± 0.19***	54.71 ± 0.31***	51.56 ± 0.07*	48.94 ± 1.19
PUFA	31.67 ± 0.36	27.66 ± 0.03***	26.48 ± 0.45***	26.17 ± 0.05***	28.97 ± 0.74**

EV, empty vector; SBP1-1 and SBP1-2, transformants with overexpressed SBP1; SBP2-1 and SBP2-2, transformants with overexpressed SBP2.

Values are mean ± SD (n = 3). Student’s *t*-test was performed to determine statistically significant differences: *p < 0.05, **p < 0.01, and ***p < 0.001.

**Table 3:** Fatty acid content of *Yarrowia lipolytica* with overexpressed SQUAMOSA promoter-binding protein.

Fatty acid (mg g^−1^)	EV	SBP1-1	SBP1-2	SBP2-1	SBP2-2
C16:0	2.41 ± 0.03	2.41 ± 0.09	3.09 ± 0.13**	3.05 ± 0.03***	2.91 ± 0.15**
C16:1	2.10 ± 0.07	1.82 ± 0.10*	2.72 ± 0.06***	2.09 ± 0.00	2.09 ± 0.01
C17:1	0.37 ± 0.01	0.51 ± 0.02***	0.47 ± 0.02**	0.62 ± 0.01***	0.45 ± 0.03*
C18:0	0.29 ± 0.01	0.42 ± 0.04	0.40 ± 0.02**	0.83 ± 0.02***	0.68 ± 0.04***
C18:1	5.07 ± 0.06	6.44 ± 0.21**	7.49 ± 0.33***	6.83 ± 0.06***	5.85 ± 0.10***
C18:2	4.77 ± 0.08	4.46 ± 0.15***	5.14 ± 0.08**	4.80 ± 0.04	4.89 ± 0.16
Others	0.47 ± 0.02	0.49 ± 0.03	0.45 ± 0.00	0.58 ± 0.01*	0.62 ± 0.01***
SFA	2.89 ± 0.04	3.01 ± 0.12	3.69 ± 0.14**	4.12 ± 0.03***	3.82 ± 0.18**
MUFA	7.67 ± 0.04	8.92 ± 0.30**	10.82 ± 0.39***	9.69 ± 0.05***	8.56 ± 0.13***
PUFA	4.90 ± 0.08	4.58 ± 0.16	5.23 ± 0.07*	4.92 ± 0.04	5.07 ± 0.16
TFA	15.48 ± 0.06	16.55 ± 0.58	19.77 ± 0.60***	18.80 ± 0.13***	17.49 ± 0.17***

EV, empty vector; SBP1-1 and SBP1-2, transformants with overexpressed SBP1; SBP2-1 and SBP2-2, transformants with overexpressed SBP2.

Values are mean ± SD (n = 3). Student’s *t*-test was performed to determine statistically significant differences: *p < 0.05, **p < 0.01, and ***p < 0.001.

## Discussion

The *de novo* TAG biosynthetic pathway and associated enzymes, DGATs, were reported to play an essential role in TAG accumulation and altering lipid composition in microalgae (Klaitong et al., 2017); however, more and more attention was paid to lipid turnover. Here, we put forward that lipid turnover plays a dominant role in the variation of fatty acid desaturation under early nitrogen deprivation from the aspects of biochemical composition, fatty acid profile, and lipidomic and transcriptomic analysis. In addition, we verified that SBP transcription factors participated in fatty acid composition variation by overexpressing them in *Y. lipolytica*.

### No net increase in lipid content under early nitrogen deprivation

Although the synthesis of starch is more energetically economical than lipid synthesis, the energy of TAG is 2.5-fold higher than that of starch, enabling its survival under long-term nitrogen starvation (Nordin et al., 2020). Consistently, on the second day of nitrogen stress, the growth of *C. pyrenoidosa* bogged down, and the protein content dramatically decreased. Carbohydrate accumulation was a priority instead of lipid accumulation (**Figure 1**). As nitrogen deprivation continued, carbohydrate was degraded while lipid accumulated. Consistent with biochemical changes, partial enzymes involved in protein and amino acid degradation were upregulated, while enzymes participating in starch synthesis were more highly expressed, and the amylolysis was suppressed on the second day (**Figure 4**). Similarly, transcripts of starch synthesis increased immediately, while those of fatty acid and TAG synthesis increased later in *Ettlia oleoabundans* under nitrogen deprivation (Sturme et al., 2018). This indicates that other pathways instead of the fatty acid and TAG synthesis pathways contributed principally to variations in fatty acid desaturation under early nitrogen deprivation.

### The fatty acid profile indicates an active prokaryotic pathway under early nitrogen deprivation

Under nitrogen-deprived, phosphorus-deprived, high salinity, high alkalinity, high temperature, or other stressful conditions, microalgae tend to accumulate SFA and MUFA while reduce the content of PUFA, avoiding lipid peroxidation and decreasing cell membrane fluidity (Sajjadi et al., 2018). Indeed, the content of SFA increased in both 16- and 18-carbon fatty acids, while MUFA only increased in 18-carbon fatty acids (**Table 1**). Acyltransferases belonging to the prokaryotic pathway had a substrate preference for 16-carbon acyl-ACP, while those derived from the eukaryotic pathway were more inclined to utilize 18-carbon acyl-CoA (Jayawardhane et al., 2018). On the second day, 16:0 increased dramatically to 48.45% of total fatty acids, meaning lipids generated from the prokaryotic pathway could prevail. Then, it decreased on the 6th and 12th days, while 18:0 and 18:1 gradually increased, indicating the accumulation of lipids derived from the eukaryotic pathway.

### Lipidomic analysis reveals detailed variations in lipid turnover

Environmental stress triggers the accumulation of TAG accompanied by degradation of membrane lipids, which prevents photooxidative damage and supplies precursors for chloroplast lipids (Solovchenko, 2012). Generally, TAGs that are *de novo* synthesized from acyl-CoA *via* the fatty acid synthesis pathway and Kennedy pathway are the majority, while TAG from polar lipid recycling contributes little to accumulated TAG (You et al., 2020). However, the contribution of lipid turnover and the Kennedy pathway was dynamic as nitrogen stress progressed in our investigation. First, LION enrichment analysis showed that 16:0 is the most significant fatty acid on the second day, then 18:1 exceeded it (**Supplementary Figure S1**), which was similar to the fatty acid profile. Second, chloroplast-derived DAG was used to produce TAG, mainly under early nitrogen stress. Based on the 16:X at the sn-2 position of the glycerol backbone, approximately 70% of TAG was generated through the prokaryotic pathway (Liang et al., 2019). C18-DAG increased on the 6th and 12th days, indicating the high contribution of the prokaryotic pathway to early variations in fatty acid desaturation and the eukaryotic pathway to later variations.

In *Chromochloris zofingiensis*, the remodeling of membrane lipids contributed more than 30% of TAG assembly on the second day of nitrogen deprivation (Wu et al., 2021). Similarly, one-third of acyl chains accumulating in TAG were derived from pre-existing membrane lipids, and approximately 45% of the FAs in TAG were derived from membrane lipids during N-deprivation by ^14^C- and ^13^C-labeling (Young and Shachar-Hill, 2021). Some studies considered MGDG, DGDG, and PC (DGTS) as major contributors for TAG assembly (Avidan et al., 2021). However, our study highlighted the role of other diacylglycerol derivatives, particularly lysolipids.

Partial phospholipids, especially those with PUFA, could be first converted to DAG and then to TAG by phospholipases due to their identical acyl moiety. Considering the similar pattern in PC, TAG, and DAG, ^14^C-acetate labeled very long chain-PUFAs in TAG were principally derived from PC, likely using DAG as the intermediate in *Thraustochytrium* (Zhao and Qiu, 2019). The variation was similar in PG, PE, and PI, which could also be converted to DAG by phospholipases (Ali et al., 2022). Given the elevated DAG and reductions in phosphatidic acid (PA) and most lysophospholipids, phospholipase C (PLC) hydrolyzing phosphodiester to DAG could contribute more. Increased levels of phospholipids containing 16:0 and 18:1 were widely reported in plants (Higashi and Saito, 2019). They could also function as acyl donors under the action of PDAT, which hydrolyzed 16:0, 16:1, and 18:1 at the sn-2 of PC, PE, and PG for TAG assembly (Yang et al., 2022). This maintained levels of 18:1/18:2-LPC, 18:1-LPE, and 18:1-LPG.

Since 18:1 was rapidly desaturated after its incorporation into MGDG, PGD1 hydrolyzed newly synthesized 18:1 from the sn-1 position of MGDG for TAG synthesis (Young and Shachar-Hill, 2021). In this study, both MGDG and DGDG were substrates of galactolipase such as PGD1 and HIL1, causing dramatic increases in 16:0-MGMG/DGMG (**Figures 3C, D**). In *C. reinhardtii*, the decrease in the major plastic MGDG 18:3/16:4 was mirrored by increases in DAG 18:3/16:4 and TAG 18:3/16:4, indicating that newly accumulated TAGs were formed *via* direct conversion of MGDGs to DAG backbones then TAGs (Young et al., 2022). However, MGDG and DGDG with PUFA in nitrogen-depleted *C. pyrenoidosa* could be converted to lysogalactolipids or glycerol-3-phosphate and fatty acids instead of DAG due to the different fatty acid compositions in DAG and TAG with above desaturate galactolipid species (**Figures 3A, B**).

As for the metabolism of lysolipids, on the one hand, they could be re-acylated to phospholipids and galactolipids by lysophospholipid acyltransferase, which could also use MGMG as a substrate (Iwai et al., 2021). On the other hand, phospholipase A (PLA) could hydrolyze the only acyl moiety; some PLAs like AtLCAT3 could also act on LPC (Richmond and Smith, 2011). They also could be converted to MAG because of the significant increase in 16:0-MAG on the second day (**Figure 3A**).

Lipidomic analysis revealed detailed variations in glycerolipid species with different desaturations, highlighting the predominant role of lipid turnover in the variation in fatty acid desaturation under early nitrogen deprivation and the Kennedy pathway in later variations.

### Multiple genes involved in lipid turnover are upregulated under early nitrogen deprivation

The *de novo* biosynthesis of fatty acids and its partition to TAG from the eukaryotic pathway or lipid turnover under nitrogen deprivation is energy and resource consuming, requiring extensive cell investments. Therefore, we investigated the expression of enzymes involved in lipid metabolism and energy supply.

Most genes involved in fatty acid synthesis exhibited no difference except for the upregulated OAH and AAD (**Figure 4**), whose overexpression promoted the accumulation of 18:1 (Liu et al., 2012; Cao et al., 2014). However, the enzymes involved in acetyl-CoA and NADPH supply, such as PK, ME, and ACL, were all downregulated, while only two subunits of PDC were upregulated (**Figure 4**). It is generally accepted that acetyl-CoA supply determines the rate of fatty acid synthesis (Sun et al., 2019). Therefore, restricted acetyl-CoA supply indicated limited carbon flow in fatty acid synthesis. On the contrary, many genes participating in the competing starch synthesis and partial genes participating in protein degradation were more highly expressed, which showed that most carbon flows were sunk in starch at the early stage of nitrogen deprivation.

The Kennedy pathway is considered the main contributor to TAG synthesis (Nagappan et al., 2020). However, three enzymes involved in TAG assembly of the Kennedy pathway, including GK, LPAAT, and PP, were downregulated, while only one of DGATs was upregulated (**Figure 4**). This meant that DAGs were mainly originated from membrane lipid turnover instead of *de novo* synthesis, demonstrating that the Kennedy pathway contributes little to TAG synthesis and variations in fatty acid desaturation. On the contrary, many enzymes participating in lipid turnover exhibited higher expression. PDAT exhibited both acyltransferase activity and lipase activity to extensive lipid species (Kong et al., 2018), and upregulated PDAT contributed to the transfer of 16:0 and 18:1 from membrane lipids to TAG. Phospholipases catalyzed the hydrolysis of membrane lipids, which could be grouped into acyl-hydrolyzing phospholipase A (PLA), head group-hydrolyzing phospholipase C (PLC), and phospholipase D (PLD); some were also active on glycolipids (Ali et al., 2022). Although the annotation of phospholipase in *C. pyrenoidosa* was indistinct, phospholipase A, patatin-like phospholipase, and other lipases were upregulated. Similarly, one PGD homologue, 18 lipases, and 3 patatin-like phospholipase genes exhibited upregulation in nitrogen-deprived *C. zofingiensis*, indicating their active involvement in recycling fatty acids from membrane lipids to TAG (Liu et al., 2019). In addition, upregulated PCYT1, and stable MGD and DGD, maintained the generation of membrane lipid species with 16:0 or 18:1, reducing the fluidity of the membrane and functioning as an intermediate for TAG synthesis (Peng et al., 2019). Therefore, we propose that the contributions of lipid turnover to variations in fatty acid desaturation under early nitrogen deprivations were greater than that of the Kennedy pathway.

### SBP transcription factors participate in fatty acid composition variation

SBP transcription factors are rarely reported to participate in lipid metabolism. Only the NRR1 was characterized as a specific response factor to nitrogen deprivation with a role in TAG accumulation (Boyle et al., 2012). In our study, overexpression of SBPs decelerated the growth of *Y. lipolytica* (**Figures 5E, F**) and changed the fatty acid profile. The percentage of 18:2 decreased in all transformants, 18:1 increased in SBP1-overexpressing transformants, while SFA increased in SBP2-overexpressing transformants (**Table 2**). The underlying causes of altered fatty acid desaturation were variously increasing degrees of absolute 18:1 and total fatty acids contents (**Table 3**). Simultaneous upregulation of SBPs and genes involved in fatty acid metabolism were detected in *Haematococcus pluvialis* under high light stress (Du et al., 2021). It was reported that SBPs bond to the “GTAC” core sequence (Shao et al., 2019), which existed in the promoter sequence of *oleoyl-thioesterase* and *fatty acid exporter* (**Supplementary Data 3**). As VuNACs from cowpea bound to “ATGCGTG” motif shared by several native transcription factors of yeast to improve its growth and stress tolerance (Srivastava and Sahoo, 2021), SBPs could upregulate transcripts of lipid metabolism to affect fatty acid profiles in yeast. However, the mechanism of SBP’s participation in regulating fatty acid desaturation requires further investigation.

## Conclusion

Nitrogen deprivation triggered dramatic variations in biochemical composition and reduced fatty acid desaturation in *C. pyrenoidosa*. We elaborated the accumulation of lipid species with 16:0 or 18:1 and the transfer of PUFAs from membrane lipids to TAG, highlighting the predominant role of lipid turnover under early nitrogen deprivation and the role of Kennedy pathway during later periods. Transcriptomic analysis revealed that intensive lipid turnover instead of an attenuated Kennedy pathway contributed mainly to variations in fatty acid desaturation under early nitrogen deprivation. We also verified the role of SBPs in promoting fatty acid accumulation and reducing fatty acid desaturation in *Y. lipolytica*. Our results help to illustrate the foundation and mechanism of changes in fatty acid desaturation, providing new targets for the modification of fatty acid desaturation in microalgae to produce high-quality biodiesel and bioproducts.

## Data availability statement

The datasets presented in this study can be found in online repositories. The names of the repository/repositories and accession number(s) can be found below: BioProject number PRJNA855982.

## Author contributions

RW and XM conceived the study. RW performed the experiments and data analyses. RW wrote the manuscript with contributions from XM who supervised the project. Funding acquisition, XM. All authors contributed to the article and approved the submitted version.

## Funding

This research was sponsored by the National Key R&D Program of China (2021YFA0909500). It was also supported by the Natural Science Foundation of Shanghai (No. 20ZR1426600).

## Acknowledgments

We thank Hairong Cheng (Shanghai Jiao Tong University) for providing yeast strain and plasmid and Dr. Feng Lei and Xin Li (Instrumental Analysis Center of Shanghai Jiao Tong University) for their excellent technical help.

## Conflict of interest

The authors declare that the research was conducted in the absence of any commercial or financial relationships that could be construed as a potential conflict of interest.

## Publisher’s note

All claims expressed in this article are solely those of the authors and do not necessarily represent those of their affiliated organizations, or those of the publisher, the editors and the reviewers. Any product that may be evaluated in this article, or claim that may be made by its manufacturer, is not guaranteed or endorsed by the publisher.
