# Supplementary figures and images for: Lipid turnover and SQUAMOSA promoter-binding proteins mediate variation in fatty acid desaturation under early nitrogen deprivation revealed by lipidomic and transcriptomic analyses in Chlorella pyrenoidosa
Source: Front Plant Sci. 2022 Sep 29;13:987354. doi: 10.3389/fpls.2022.987354 (PMC9558234; doi:10.3389/fpls.2022.987354)

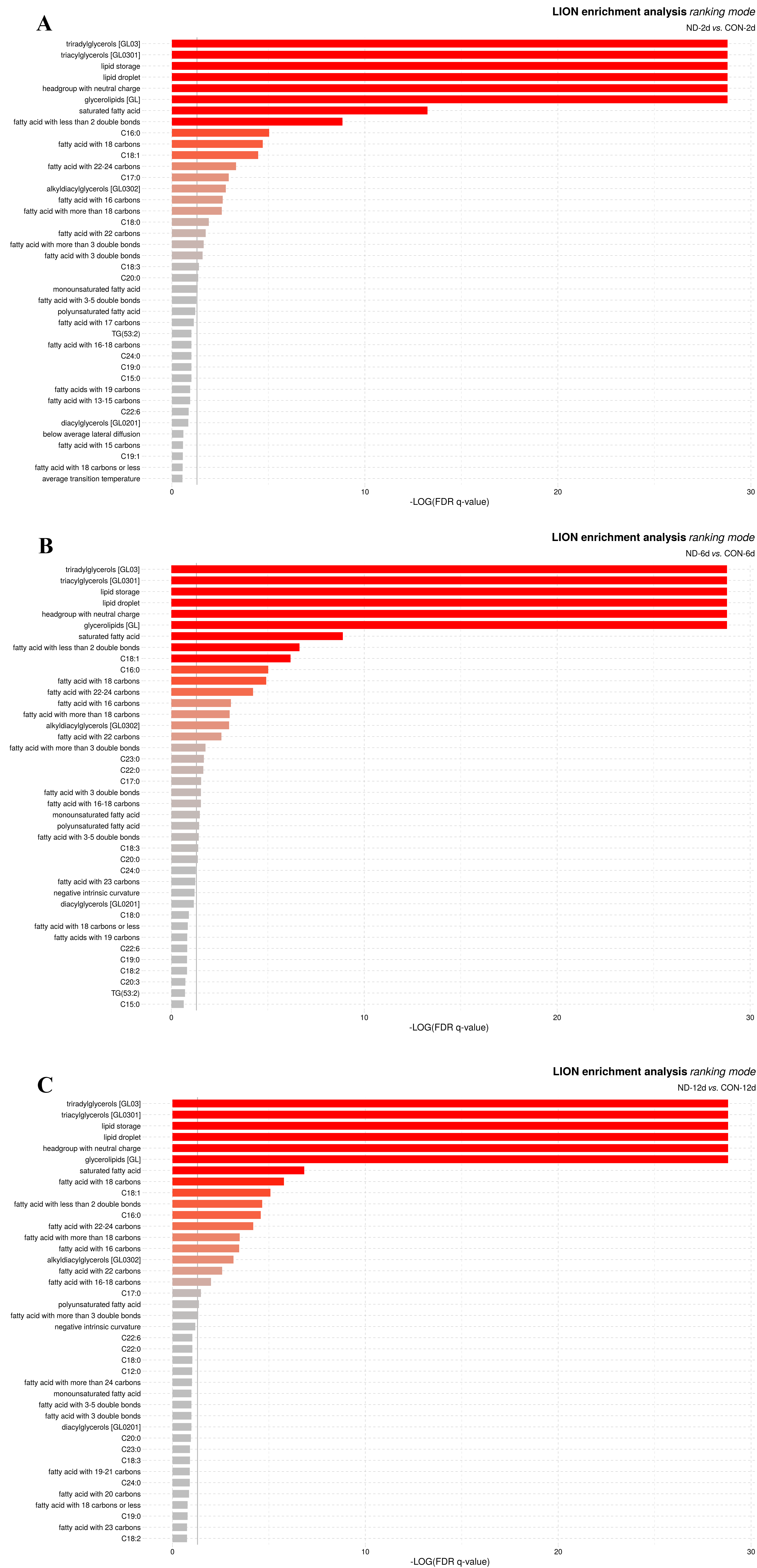

Supplement: Supplementary file 1 [file Image_1.tif]

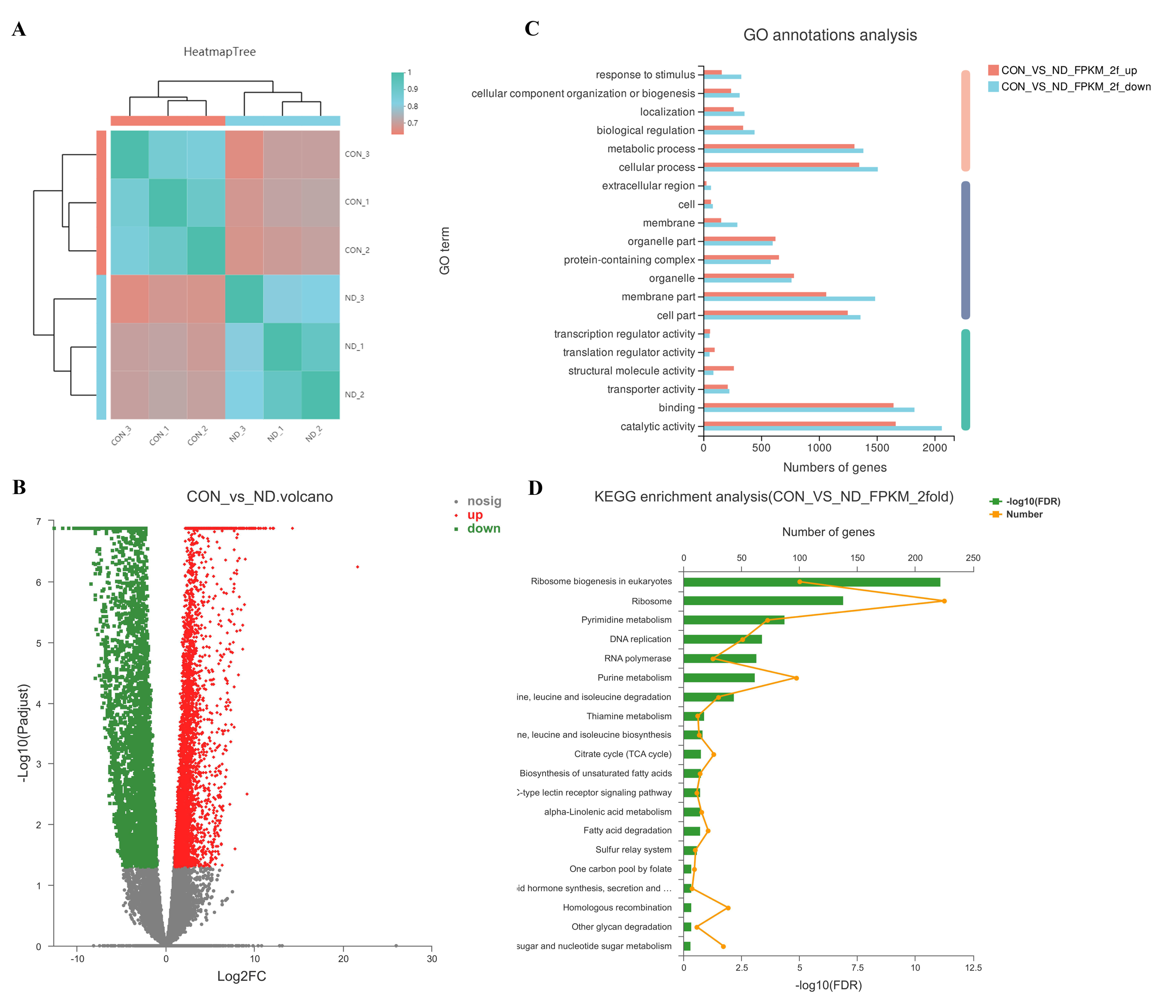

Supplement: Supplementary file 3 [file Image_3.tif]

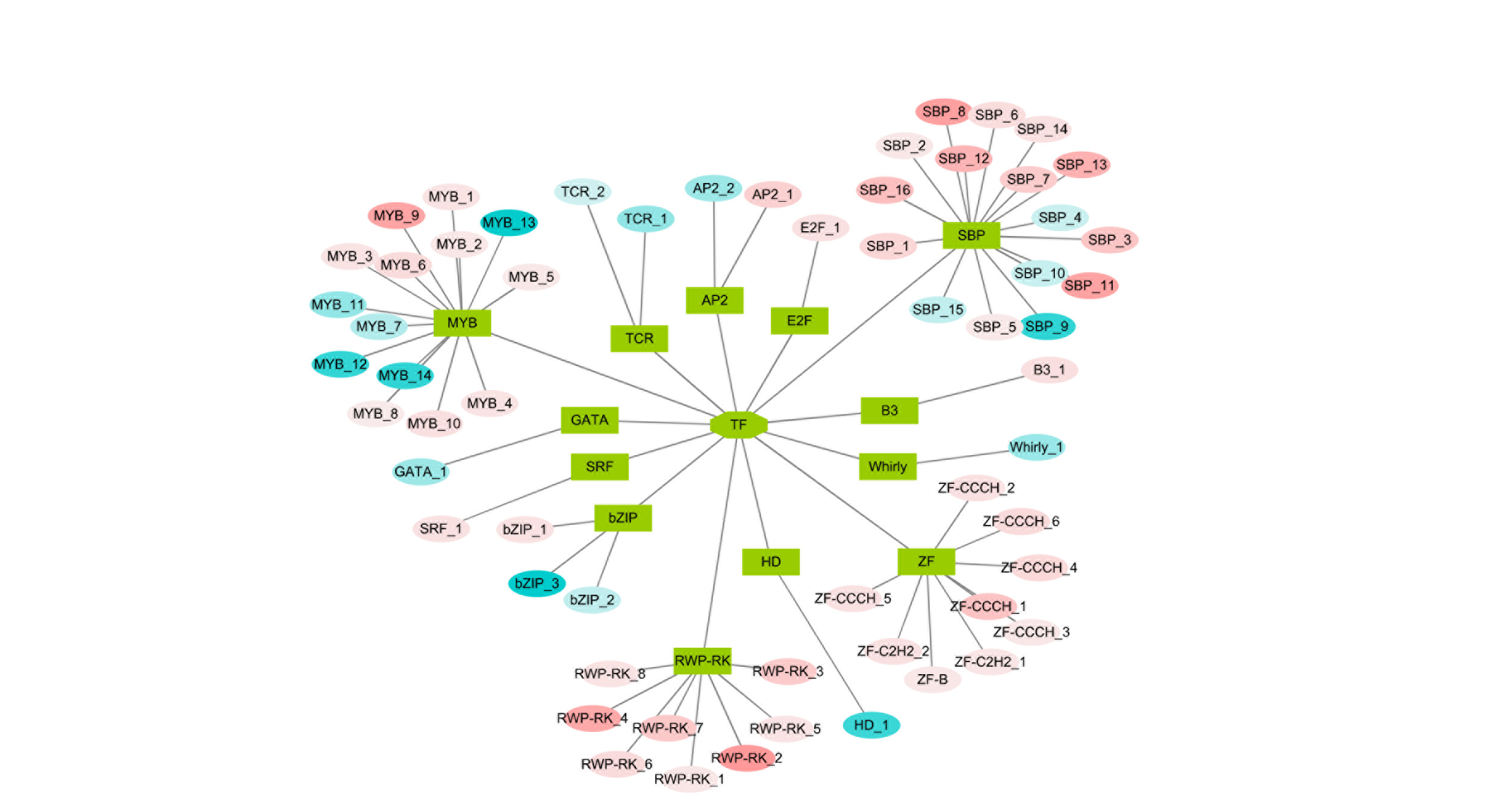

Supplement: Supplementary file 4 [file Image_4.tif]
