# Supplementary material for: Lipid turnover and SQUAMOSA promoter-binding proteins mediate variation in fatty acid desaturation under early nitrogen deprivation revealed by lipidomic and transcriptomic analyses in Chlorella pyrenoidosa
Source: Front Plant Sci. 2022 Sep 29;13:987354. doi: 10.3389/fpls.2022.987354 (PMC9558234; doi:10.3389/fpls.2022.987354)
Supplement: Supplementary file 7 [file DataSheet_3.docx]

> TRINITY_DN3848_c0_g2 (SBP1)

ATGCAGCCGTCAGCCAGCGGCGGCACGGCTGTAAAAGGCTGGGAGGGGCCGGAACATGGCGCCAAGCCCCCTGCCGGTGGTGGTGGCAGGGCCTCGAGAGCGCAGTGCAGGGTTGAGGGCTGCACAGCCCCTCTGCAGCAGGCCAAGCGCTTCTACCAGCGGCAGCACATTTGTGAGAGGCACTTCCAAGCGCTCAGCATCACCGACGCAAACGGCCGGCGGCACCGCTTCTGCCAGCAGGCGAGCAGCTTGCTTGCTTGTTTGGGCTTAAAGTCTGTCTATCCACAACAGAATTTGCGACACAGACCCAGTGCACCCGCCTGCACCCCATTTCTGAGTTTGAGGGCACAAAGAGAAGCTGCAGGAGCAGCCTCGCTCGCAGACAGGTGGGCTGGACAGGGGATGGAAGCAAGGAGGAGGTGCGAGGGAGAGAGGTCTTGGACTGGTTGTTCTCAGAAACGAGCACCTGGCTGTGCTCAGAAACAAGCATCTGGCCGCTAG

> TRINITY_DN9157_c0_g1 (SBP2)

ATGGCGGCAGACAGCGAGCAGCTGGATGCCGCACAGTCTGCAGGGGGACTGGCATCCTACTTTGCGGCAAGAGCTACTGCAGGTGCTGTGCAGGCGCCTGCAGCCGGTGGTCAAGCAGCCGACAGGCAGCCAGAGGGGCCGCAGCGGCGGGGAAGGCGCGGCCGGCTGCCCCGGACCTCAATCACCTGCCAGGTGGAGGGTTGCAGCGCCGAGCTCATTGGCGAGAAGAGATACTATCAAAGATATCAGATCTGCCCGCCGCACTGCCGCATGGATCGCCTGGTGGTGGGTGGAGTGCTGCAACGCTTCTGCCAGCAGTGCGGTCGCTTCCACGTGCTGGCGGAGTTTGAGGGCACAAAGAAGAGCTGCGTCCGCAAGCTGCGGTTGCACAATCAGCAGCGGCTGCAGGGCACTCGAAAGGCAGCTGCAGAGGCTGGCGCCGCTCAGGCACAGCAGCAGCAGCACCCTCCTGTGGGCAAGGGCCCCCCGGCTGGCCTCTGGCAGCTCCAGCCGTGCACAGCAGGGTCCAGCAAGGGCCATATGAGTGGACGCGCCTGTGCCCAGGGAAGCGCAAGCCACGGCGCTCCTTCAGCAGTCGTCACAAGTGGACTGGGCGTGACCGCAGGCTCCTGGGCCGAGGCGCCAACACGCCAGCATCATGCAGCGGTGGCGGCGGCTGGACCGAGGCTCATTCTGCATGGAACGCCGGCCAAGACGGACCCAGGGATCACGCAGGCGGCAGCGGCGCAGCCACATGCCGCTATACCCTTGGCGCCTTCAGCGATCCGCCCTGCAGACCTCGCAGCTGCGCGGCTGCCGCCGGCGAATCCAGCGGCGCCCTTAGCAGCGCCACACGCAGTTGCAGCACCAGGTCCCCCCCTGCAACCTTCTTCTGCTGGCAGCCGGACGACAGCTGTTGTGGGGGCTCCCATTTCTGAAGCACACGCCGCACAGGCGCTGGCAGAGGAGCTGTGCAGCATGCTCGGGACGCCCACCCCGGCGGGTGCATCAGCTGATGCTGGGATGCAGGGCGCGCTCCTCCACGGGCTTTCCAGCACTCTGCTGGTGCTTGCAGCGCTGGCGCAGGCATGCGGCACGGAGAGCATTGTGCATGCGCAGCAGGCAGAGCAGCAGGTGGGGCAGCAGGCACGGCGCGGAGAGCAGCTAGTGGAGCGGCAGGAGGAGCACCAACAGGAGCAGTAG

> Up-stream sequence of *oleoyl-thioesterase* -2366bp

ATTGGCACGATTGGAAAGTTCGGCGAACTCGACATCGAGGGCAAGAAGCTCTGCCTGGTGAGTCCCAAGAACCTACAAGAACCAGTTTCAGCTGAATCCAGCTCGTGTGCCATCCGCTGGGTTTGCGATTTCAGCACGTGTGGGCCCGCTGGCCGTGCTAACAGCATGCGCTCTCTTGTTTGCGCCATGATGCCTCCCTGGCTGCACCCGCTCGTGTCTTCTTCACGCCGCAACCCCCAGCCCGGTCCGGCCAGGAGCGCTTTGAGCGGGTGGCGGAGAAGCTCAAGGTGATCATGGCGCGGGTGCGGCTGGCCAAGGACGACCCGCTGGGCCAGAAGATGCTGCGGCTGCAGGCGGTGCATCTGTGAGCGGGCGGGGCAGGGCATGCGCTGCCTGCAGGCAGGCGCGTGCCGATGCCGCGGGCACCTGGCAGGACGTGCAAGGAGGTGCCGCACGGCCCAAGCGCCTGGCACCCGTACCCTGAAGCTAAGCAATGCGCGTTGCTGATGGCATTCCCGGCCATCTGGCATGGCCGGCCATCATCAGCGCTGCTGTGCGTTCCTGCCTGCCCGTCTGCCGACGGGATGGGAGCCTGGGTGCAGGGAGCGCGCATGGGGGCAGCGCCTGCATCGAAGCTGCGCTTTGGGGGCCCTGTGTGCAGGCTGGAGGCCAACACCAACCTGGACATGATGCTGCAAGGGTGAGGTATCGCGCTGGTATGGTGGGCCAAAAGGGTTGAGTTGGCCTGCCAGGAGCGGGGGGGCATGTGGGCGACGCCGCCGCACAGGAAGGCCTGCGCTTTGCCTCATGCCGAGCAGACCCCTTGAGCCCTCGGCCTGTACGGCTCACTGCAGCATGGATGCTGGGGTGGCAATCATGCGCAGCATGGTTGCACAGGAGGAGGCGACCCACGACCCCGCTGTGCTGGCAGCCATCAAGCAGGAGTGGAGCAAGCAGGTGTGAGGAGGGCCGGAATCGCCGCGATGCTGATGGCTGCTGTGTTGTTGCCAGCCAGCTGGCACAGCTGGACTGCGCGATGTCATCGTGCCAACCAGCCTGCATTGCCACTGCTTCCCACGCCCGCGGGCATTTCTTGACGCAGCCTGCCTCGCAGCTCAGGACACACGCTGGCCTTCCCCGTGCCACCAACCTGCGACCCAGCTCTGCGCCGCCTGCGCCCACACCCCTGCCTGCCTTCCCAGCAGTTCGGCGTGCTGGGGGCCATGAACATGGATGCAGTGATGCGCGACCCGCTGGCGGCTCAGGGCTCCATGGACCCGCTGGTGGGTGCTGCGCGGGCGTCGCCAGCTGGCAGCGCGACCAGCTGACATCGGGGCGGCACGTGCGAGTGCAGGGGAGCATGCGCGTAGGGACACCATGCCAGCACACGCCGGGCGGGCGGTGCTCGCGCTGGCCTGGCCCCTCCTGCCTGCGAGCCCTTTCACTCTCAGAGGAGCGCAAAGACGTGGAGGCTCATGCAGGGGCCCTGGCATGCTTGGCGTCCCCCCCTAACCGTTGCTGCCTGCTGGGTGTTCACCGGCACGCTGTCCTGCCCAACCCTCGGCGACGCAGGCGCTGAAGGCCGGCCGCGAGATGATGGAGAATCCGCTGGCAGTGGAGAAGCACCGGGACAATGCGCCGCTGTATGCCTTCCTCAAGTGCATGGTTCTGGGCCAGCATCCGTGATGGCGTGTGCTGCGCTGGGGATGCCGCATGGCCATTGCTGTGGTGCGGTGCGCGTGTTGCAGCAGCAATCCGCGTGCACCACCACTCCCCACCCGACTGAGCCACCTGTAGTCGACCCTGCCTGCTGAGCTGCTGCGCGATGTAAAGAATGGAGGGAGGCGAGACAGAGGCGGCTGCAGGCGGCCTGCGAGCCAAGCTGGACATGATCCTCGACCGGCAGCCGTGGCGGCATGACAGCTTGCTCAGTGCATTTACGTGCTACTAACGAGCACTCTGCAGGGAAGATACATGATGTCAACTGGCAATGATGCAATTGCCACAAGGTTTGAAAGGGGTTGTTTGAAACGTGCACGTGCCCGCAGCAGCATGTTCGGCGGCTTGGCTTCACCACCAGCCGTGTAGCCCGCAGAGTAAGTTTGCACGCGGCCATCAGTGCTTTGAGATGATCACGTGTGTTTGCGAGGCTCACGGCACCGTCACTCGGCCCGATGCCAACAGGCCTTTGGCGTCAACGCTCCGCAGGCGCCGCATGACCCACAAACCGCAAGCTTCCGCTTTCCCCATCTGTAACTGCAGCGCCTTTCGACATCGCTCCTCCAAGGTCACCGCCATCAAGCCTGCCTCCGCATGGCCATCTCCTGTTGAGCGCCACACCCTTCCTCAATTGTGCAAAGCTCAGT

> Up-stream sequence of *fatty acid exporter* -2575bp

ATAGCAGAAAGTATCCCAGGTTGAGCGCAAACTGGGCGCTGGCCAGGCCCACGATTGCGCCTGCGCCAGGGGCAGGGGAGGCAGGGCAGCAGGCAGGCGGCCCAGGTCACACGGTGAGGGGCCACACTGCCGGGGAGATTCGACCAGCTCAGGGTGGAGCTCGTTGCTGCCAGCTGCTGGACAGGGGCCGCAGGACTCACAGCCAACCTTCCCAAGTCAAGAGCCATAGGTGCAGAAGGGAACTGCATGCGGCAGGCGTCATCCAGGGGCACGCGGGCACCCGCTCACCCGAGGCGCTGCAGTTGTAGGTGGCGGTGTGGCATGCCGGCGGAGGTGGCACCTGCTGCCCCCCGACCTTGGTCTCCACCACGGCCGACAGGGCCGCATAGTTTGCCTGCACCAGGGCCCAGTAGAAGCCTTTCTTCCAGTGCCACCACAGCGGGGCGTCGCCGACTAGCGGCGTGCCGCGCTCCCCGGGCCGCTCCACGCCTGCCGCCATTGAGGGGGGGGCAGCGGGGGATTGATGTAAGCTGTGCCTGCATGCTTGCCATGCAAGGAACACAGCGGAGTCCAGCCTGCTGAAATGCTGTCAGCAGCAGCCGGGCTGTGACCTGCGACGACGGCCCTGGGCTGGGCTGACTCACGCCAGCTGCCCCGCGCCGGGTTGCCGCTGTGTGCCTCCACCAGGTACAGGCAAAACGTGGTGTTCCAGCAGGTCCACCTGGGGAGGGGGGGGGGGTTCGTGTTGGTGGTGGTGGTGGTGGTGGGTCTCAGCAGAGGTGCAAGCAGCATGCTGGGGGCACGGCCAGTTGTTGCGGCGGGGAGGCTCAAATGCGGACCATGGAGATCCATGCAGTGAAGGCAACATGAAATACAGCCCAGCCCGTGCTCAGCAAACGGCTAGACCCCCAGCTGCAGAGTGTCTGCATGGCAGCTTGGGTGGCGGCGCTGCATGCCATCTGGGCAGGCGACCGAGAGCTGGCCAGCGCCCACCTCACAAAGGCGCTCCAGGCGATGAGGGAGCTGAACCAGGTGCATTCGTGCACTATTAGGGAGGCATTGGGTGCGAGGAACAGGCAGGCACTGACCAGCTGCAGCGCCAGCTCGGCCCCCGCCAGCGTGGCCAGCCGCCGCCGGCGGTGCGACCAGCGCTTGCCGCTGGCATTCACCCTGCAGATGGGCGCGGAGCCTGCGTCACTGCGGCCTGTGCTGGTCGCCGCTGTGTACTGCAACATGTCCCAGTGCTGCGTGCCTGCCAGCTGGCTGGCCCACCAATCCACCTGGCTTCAGCCACCTGCCTGCTCGCCAGGCCTGGCAGGTACCTGAGCACGCGGCGCAGGAAGAAGATGCCATAGGCCAGCAGCGCGGCAGTGCACAGCGCCGCACAGGCCAGGTTGGTGATAGGCAAGGCGTCGTGCATGAACAGCCATGAAGGGTCCTGGAAGGGTTTGCGCCGGTGGGGGGCAGGGCGCGCGTGTGAGGGGACGTGCAGAAGCGCGGCAGCTCGGCAGCGCAGGGAGCGCGGGCCATGCCCGGCGCACCCCCTGGCACCTTGCTCGCTCAGCCCCACCTGGCTCTCCTGTACGTAGACCACCGCCACCAGCAGCGCAACGCCCAGCAAGATCACCGCCCACAGCAGGGCAATCACGATCTGCGCAAGACAGGCAGGAGGCATCGGTGCCAGGCCTGTGCCGATGAGCCCAGCCTTGAGGCTGGCCGTACCAGTGGTCCGGCTGGCGCCAGCGTATAGGTCGAGCACTTCATGTGATCACGATCATGCGCAGCATGTGCAGCAGCATCCAGGCATGTCTGCAGTGCCAGACTGGCAACGTGGGCTGGCAGCAGCTCCCCAGTCGGCCCTGCTTCACGCCACCCACCTTGACAGCCCTGCTGGAAAGCACATCATAGCGCAGCACCACGTCCCTCGCCGGATCATAGTGCGGCGTCAGCGAGCTGCGCCCCGACAGCTGCGGCTCCAGGGCCTGCTCGGCATCGGGGCCACCGCCCTCAGGGAAGCCACCTGCATCCGCCCCTGCATCACCTTTCTGTGTGCTGCCTGCGGCGGTGGTCACAGCGCTCCCATCCTCTTCCTCCTGGCAGCGCTGCATCATCCCTACGTGGCACTCTGTGCATTACGCATTGCCTGCCTCCGTCGCACAGGACCTGCACGGCGGCAGCGGCGGCTGCGGCACCCTGGCTGTCAAGGCCAACCGTACTCTGCGCTGCGCGGTGTAGCAGTAGATATGCTGAAATGGTATGAGCTGCCTGTTCACTGCCAAACGGCTGCCTGTCACTGCTGCAGCTGGTTGTGCAGCAGAATAAGCGCGCACGCGCTATCCTTTGATCACCTCACGACATGCGCGAGCATGCTGCCTGCACGAGACCCCTTTGGAAGGGACAGTCGCATCCCACCCTGCTGTGCGCACGCATCACGACCCTTGTATCTCGCGGGATGTTGACGGTCGGTGTCTCCTGGCGCAAAAATCTGTGCAGCACTTTGTGTACATCGGAGCACCACTCCACCCACCAGCCCAGAGGCACCCCCGCTGCGTCGGTCCTTGGGGCAGCAACAGCT
