# Supplementary material for: Lipid turnover and SQUAMOSA promoter-binding proteins mediate variation in fatty acid desaturation under early nitrogen deprivation revealed by lipidomic and transcriptomic analyses in Chlorella pyrenoidosa
Source: Front Plant Sci. 2022 Sep 29;13:987354. doi: 10.3389/fpls.2022.987354 (PMC9558234; doi:10.3389/fpls.2022.987354)
Supplement: Supplementary file 8 [file DataSheet_4.docx]

Supplementary Material

**
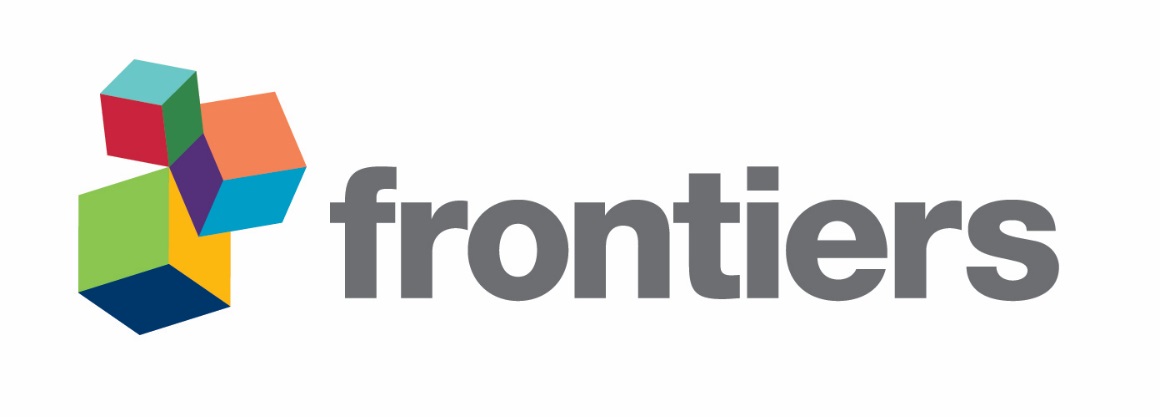
**

**Supplementary Figure 1.** Enrichment analysis of lipids in the “ranking mode”. **(A)** Nitrogen deprivation (ND) vs control (CON) at the second day. **(B)** Nitrogen deprivation (ND) vs control (CON) at the sixth day. **(C)** Nitrogen deprivation (ND) vs control (CON) at the twelfth day. Gray vertical lines indicate the cut-off value of significant enrichments (q < 0.05). Bar colors are scaled with the enrichment (−log q-values). FDR: false-discovery rate.

**Supplementary Figure 2.** The box-whisker plots of all lipid classes. MG, monoacylglycerol; DG, diacylglycerol; TG, triacylglycerol; MGDG, monogalactosyldiacylglycerol; DGDG, digalactosyldiacylglycerol; SQDG, sulfoquinovosyldiacylglycerol; MGMG, monogalactosylmonoacylglycerol; DGMG, digalactosylmonoacylglycerol; SQMG, sulfoquinovosylmonoacylglycerols; PA, phosphatidic acid; PC, phosphatidylcholine; PE, phosphatidylethanolamine; PG, phosphatidylglycerol; PI, phosphatidylinositol; PS, phosphatidylserine; LPA, lysophosphatidic acid; LPC, lysophosphatidylcholine; LPE, lysophosphatidylethanolamine; LPG, lysophosphatidylglycerol; LPI, lysophosphatidylinositol; LPS, lysophosphatidylserine.

**Supplementary Figure 3.** Expression analysis of differentially expressed genes in *Chlorella pyrenoidosa* under nitrogen deprivation. **(A)** Heatmap of sample correlation coefficient. **(B)** Volcano-plot of differentially expressed genes. **(C)** GO annotations analysis of differentially expressed genes. **(D)** KEGG enrichment analysis of differentially expressed genes.

**Supplementary Figure 4.** Network of differentially expressed transcription factors (TFs) under nitrogen deprivation. The network was analyzed and visualized using CYTOSCAPE. Red nodes indicate up-regulation, and the blue nodes indicate down-regulation, colors are scaled with the fold change.

**Supplementary Data 1.** Response area of glycerolipid species under nitrogen repletion and deprivation conditions at the second, sixth and twelfth day.

**Supplementary Data 2.** The mRNA-Seq data for the genes involved in carbon metabolism, nitrogen metabolism, photosynthesis, acetyl-CoA and NADPH supply, protein degradation, and signal transduction.

**Supplementary Data 3.** Sequence of SQUAMOSA promoter-binding proteins as well as up-stream sequence of *oleoyl-thioesterase* and *fatty acid exporter*.

| Primer | Sequence |
| --- | --- |
| 1560_qPCR_F | CTACAGCATGGTCTCCGTCC |
| 1560_qPCR_R | CGTGCCAAACACAATCAGCA |
| 5070_qPCR_F | GTGGAGGCACACAGGATTCG |
| 5070_qPCR_R | CGACATGGAAGGGTCAGAGG |
| 19530_qPCR_F | CACGGGCATTGAGACCAAGG |
| 19530_qPCR_R | TGACGGTGACGCACTTGTAG |
| 12146_qPCR_F | CTTCCATACGACCACCTCGC |
| 12146_qPCR_R | GTCACCAAAATGCCAGACGG |
| 6685_qPCR_F | TGCAGGCAGAGTTCCCTATG |
| 6685_qPCR_R | GCTCGGAAGCATCTTGGAGT |
| 23676_qPCR_F | GGAAGCGTATGAGGATCGCA |
| 23676_qPCR_R | CTGGTAGGAAGGTGGCGATG |
| 24789_qPCR_F | TTCTTCGAGAGCTTCCGCCT |
| 24789_qPCR_R | GTGCGCCTTCTTCCACTCTC |
| 8962_qPCR_F | GACGGTGCGTTTTGACTACC |
| 8962_qPCR_R | CCCTGCAAGCATCTTTTCCC |
| 12225_qPCR_F | CTCGCTGCCTCAAGCTCTAC |
| 12225_qPCR_R | GGGTCTAAAGGCATTGGGGT |
| 2896_qPCR_F | CCCAAGTTTGTGTCGCCCT |
| 2896_qPCR_R | GCGTGTATTGCTCTGCTTGG |
| 677_qPCR_F | TTTACACGAATGGAGGCGGC |
| 677_qPCR_R | CGGGATTTTGTCCTCGGTGT |
| 278_qPCR_F | AACCCCTACCTGGGCTTCATCT |
| 278_qPCR_R | ATGCGGGTGTAGGCAATCTCG |
| 850_qPCR_F | CAAGGACGAGGTGTTTGTGCC |
| 850_qPCR_R | GGGAGGAGACGCTGAAGAAGAG |
| β_Actin_F | GCTCAACTCCTCCACGCT |
| β_Actin_R | GTCCTTGCGGATGTCCAC |
| pINA1313-F | TTCGGATCCGGTACCTAGGGTGTCT |
| pINA1313-R | TGTGGATGTGTGTGGTTGTATGTG |
| homo-SBP1-F | CCACACACATCCACAATGCAGCCGTCAGCCAGCGGCGG |
| homo-SBP1-R | GGTACCGGATCCGAACTAGCGGCCAGATGCTTGTTTCTG |
| homo-SBP2-F | CCACACACATCCACAATGGCGGCAGACAGCGAG |
| homo-SBP2-R | GGTACCGGATCCGAACTACTGCTCCTGTTGGTG |
| SBP1-qPCR-F | CTGTAAAAGGCTGGGAGGGG |
| SBP1-qPCR-R | AGCCCAAACAAGCAAGCAAG |
| SBP2-qPCR-F | ACAAAGAAGAGCTGCGTCCG |
| SBP2-qPCR-R | ACTCATATGGCCCTTGCTGG |
| YL-actin-qPCR-F | TCCAGGCCGTCCTCTCCC |
| YL-actin-qPCR-R | GGCCAGCCATATCGAGTCGCA |

**Supplementary Table 1.** Primers used in this study

**Supplementary Table 2.** RT-qPCR analysis of differentially expressed genes in *Chlorella pyrenoidosa* under nitrogen deprivation.

| Gene ID | Annotation | log_2_FC | -ΔΔCT |
| --- | --- | --- | --- |
| TRINITY_DN1560_c0_g1 | Acyl-CoA synthetase | -3.83 | -3.41 |
| TRINITY_DN5070_c0_g1 | Acyl-ACP thioesterase | 2.55 | 4.68 |
| TRINITY_DN19530_c0_g1 | Beta-hydroxyacyl-ACP dehydrase | -4.03 | -0.32 |
| TRINITY_DN12146_c0_g1 | Triacylglycerol lipase SDP1 | -4.66 | -2.00 |
| TRINITY_DN6685_c0_g1 | Myb DNA-binding transcription factor | 1.58 | 1.08 |
| TRINITY_DN23676_c0_g1 | Myb DNA-binding transcription factor | 1.39 | 1.33 |
| TRINITY_DN24789_c0_g1 | Myb DNA-binding transcription factor | -7.40 | -6.74 |
| TRINITY_DN8962_c0_g2 | Zf-CCCH transcription factor | 2.21 | 2.67 |
| TRINITY_DN12225_c0_g1 | Tesmin/TSO1-like CXC domain protein | -3.06 | -3.85 |
| TRINITY_DN2896_c0_g2 | Myb DNA-binding transcription factor | -3.04 | -4.61 |
| TRINITY_DN677_c0_g1 | Glutamate dehydrogenase | -5.51 | -2.04 |
| TRINITY_DN278_c0_g1 | Stearoyl-ACP desaturase | 1.56 | 3.17 |
| TRINITY_DN850_c0_g1 | Δ 12-desaturase | 2.70 | 2.69 |
